# Are *Melanoides tuberculata* and *Tarebia granifera* (Gastropoda, Thiaridae), suitable first intermediate hosts of *Clonorchis sinensis* in Vietnam?

**DOI:** 10.1371/journal.pntd.0009093

**Published:** 2021-01-25

**Authors:** Hung Manh Nguyen, Hien Hoang Van, Loan Thi Ho, Yulia V. Tatonova, Henry Madsen

**Affiliations:** 1 Institute of Ecology and Biological Resources (IEBR), Vietnam Academy of Science and Technology (VAST), Hanoi, Vietnam; 2 Graduate University of Science and Technology, Vietnam Academy of Science and Technology (VAST), Hanoi, Vietnam; 3 Federal Scientific Center of the East Asia Terrestrial Biodiversity, Far Eastern Branch, Russian Academy of Sciences, Vladivostok, Russia; 4 Parasitology and Aquatic Diseases, Department of Veterinary Disease Biology, Faculty of Health and Medical Sciences, University of Copenhagen, Copenhagen, Denmark; Chinese Center for Disease Control and Prevention, CHINA

## Abstract

**Background:**

Two thiarid snail species, *Melanoides tuberculata* and *Tarebia granifera* have been reported as first intermediate hosts of the liver fluke *Clonorchis sinensis*; however, their role as true first intermediate hosts has not been verified. Thus, the present study aimed to clarify the suitability of these two snail species as first intermediate hosts of *C*. *sinensis*. This was accomplished by collecting snails from a highly endemic area for *C*. *sinensis* in Vietnam, the Thac Ba reservoir, and identifying shed cercariae using molecular techniques. We also conducted experimental infections of five snail species including *M*. *tuberculata* and *T*. *granifera* with eggs of *C*. *sinensis*.

**Methodology/Principal findings:**

A total of 11,985 snails, representing 10 species were sampled. Five snail species, *M*. *tuberculata*, *T*. *granifera*, *Lymnaea swinhoei*, *Parafossarulus manchouricus*, and *Bithynia fuchsiana* were found shedding cercariae with an overall prevalence of infection ranging from 0.7% to 11.5%. Seven cercarial types were recorded. Cercariae of *C*. *sinensis* were only found in *Parafossarulus manchouricus*. Using a multiplex PCR approach for detecting *C*. *sinensis* infection, the prevalence in *P*. *manchouricus* was 4.2%. Additionally, all five snail species were experimentally exposed to *C*. *sinensis* eggs, however only *P*. *manchouricus* was successfully infected with an infection rate of 7.87%.

**Conclusions/Significance:**

We confirmed that in the Thac Ba reservoir, Vietnam, the two thiarids, *M*. *tuberculata* and *T*. *granifera* are not suitable first intermediate hosts of *C*. *sinensis*. Only *P*. *manchouricus* was found infected by *C*. *sinensis* in nature, and was the only species that became infected experimentally.

## Authors’ summary

Currently 13 snail species are reported as first intermediate hosts of *Clonorchis sinensis*, including two species of the Thiaridae, *Melanoides tuberculata* and *Tarebia granifera*. Both snail species have wide distributions in tropical and subtropical waters across the World, while the distribution of *C*. *sinensis* is much narrower and only occurs in endemic areas of East Asia. The role that these two thiarid snail species plays as successful hosts of *C*. *sinensis*, however, has been refuted in other studies. The present study was conducted to clarify this issue. Based on the results from field surveys in a newly discovered and highly endemic area for *C*. *sinensis*, as well as, data obtained under experimental conditions, we confirm that in Northern Vietnam, the two thiarid snail species are not successful first intermediate host of *C*. *sinensis*. *C*. *sinensis* was only detected in a single snail species, *Parafossarulus manchouricus*, through observation, molecular analyses and experimental infections.

## Introduction

One of the most important fish-borne zoonotic trematodes is *Clonorchis sinensis* (Cobbold, 1875) (Opisthorchiidae), the small liver fluke that parasitizes humans and fish-eating mammals throughout Northeast Asia [1−5]. This trematode species causes serious disease in humans and animals, including; pericholangitis, pyelophlebitis, cholangiohepatitis and multiple abscesses [3,4].

Thirteen freshwater snail species have been reported as first intermediate hosts of *C*. *sinensis* (Table 1). A majority of these determinations, 10 of the 13, are based solely on morphological examination of cercariae (pleurolophocercous type). Only two species, *Alocinma longicornis* and *Parafossarulus manchouricus*, are confirmed first intermediate hosts through experimental infection and molecular diagnostics [6,7] and an additional snail species, *Bithynia fuchsiana* has been confirmed by molecular data alone [8].

**Table 1:** List of the first intermediate hosts for *C*. *sinensis*.

Snail species	Locality [Reference]
**Assimineidae**	
*Assiminea lutea* Adams, 1861	China [9,10]
**Bithyniidae**	
Alocinma longicornis^*^^,^ ^**^ (Benson, 1842) (synonym: *Bithynia longicornis*)	China [8,11−14]
*Bithynia chaperi* Morlet, 1886	Vietnam [1,12]
*B*. fuchsiana^**^ Moellendorff, 1894	China [8,11−14]
*B*. *misella* Gredler, 1884^*~*^	China [11,13,14], Vietnam [15]
*B*. *siamensis* Lea, 1856	Vietnam [16]
*Parafossarulus anomalospiralis* Liu, Li & Liu, 1985	China [11,14,17]
*P*. manchouricus^*^^,^ ^**^ (Bourguignat, 1860) (synonym: *P*. *striatulus*)	China [8,11,12,14], Japan [1,18], Korea [1,19], Russia Federation [1,2,7], Vietnam [20,21]
*P*. *sinensis* Faust, 1930	China [12,14]
**Semisulcospiridae**	
*Semisulcospira cancellata* (Benson, 1833)	China [14,22]
*S*. *libertina* (Gould, 1859)	China [11]
**Thiaridae**	
*Melanoides tuberculata* (Müller, 1774)	China [23], Vietnam [20,21]
*Thiara granifera* (Lamarck, 1822)	Taiwan [11]

Note

* Confirmed by experimental infection

** Confirmed by molecular data; ^~^ failed in an experimental infection.

*Melanoides tuberculata* and *T*. *granifera* are highly invasive species and are currently present in North (southern USA, Mexico), Central (Panama, Caribbean region) and South America (Venezuela, Brazil), Europe, Africa, Australia, Pacific islands and Asia [1,24,25]. Mas-Coma & Bargues [1] mentioned that their wide distribution potentially could result in the global spread of *C*. *sinensis*. However, the role of thiarid snails as first intermediate hosts of liver flukes has been questioned by Bui et al. [26] and Nguyen et al. [27], as they did not find cercariae of *C*. *sinensis* within thousands of specimens of thiarid snails collected from areas known to be highly endemic for clonorchiasis in Vietnam. In addition, Besprozvannykh et al. [28] experimentally exposed fish to all cercarial types shed from *M*. *tuberculata* collected from Northern Vietnam and found no *C*. *sinensis* metacercaria. Similarly, in China, Zhang et al. [12] found *C*. *sinensis* larvae within four snail species, i.e. *P*. *manchouricus*, *P*. *sinensis*, *B*. *fuchsiana* and *A*. *longicornis*, while *M*. *tuberculata* was not infected.

The present study aimed to verify the role of *M*. *tuberculata* and *T*. *granifera* as first intermediate hosts of *C*. *sinensis* through multiple approaches, i.e. field surveys, experimental infection, and molecular detection of *C*. *sinensis*.

## Materials and methods

### Ethics statement

All applicable international, national, and institutional guidelines for the care and use of animals were followed. Euthanasia of laboratory animals was carried out in accordance with the Committee on the Ethics of Animal Experiments of Federal Scientific Center of the East Asia Terrestrial Biodiversity, Russia (Permit Number: 3 of 02.06.2011).

### Study area, snail sampling and examination

Thac Ba reservoir covers an area of 23,400 ha of Yen Bai Province in the mountainous region of Northern Vietnam. There are 12 ethnic groups living in communes around the reservoir, and local people usually prepare and eat raw fish from the reservoir [29]. According to recent studies, the Thac Ba reservoir is known as a highly endemic area for *C*. *sinensis* [29,30]. In this region, the sewage system of each household is very simple and domestic waste water and latrines flush directly into canals nearby which flow into the reservoir. Domestic animals (e.g. cattle, pigs, ducks, chicken, dogs, cats) are free roaming during the day and locked up during the evening and nights. Manure from domestic animals is likely to enter the reservoir. The coexistence of intermediate hosts (snails and fishes) and the habit of eating raw or insufficiently cooked fish explain the high prevalence of fish-borne zoonotic trematodes in this region.

**Fig 1:**
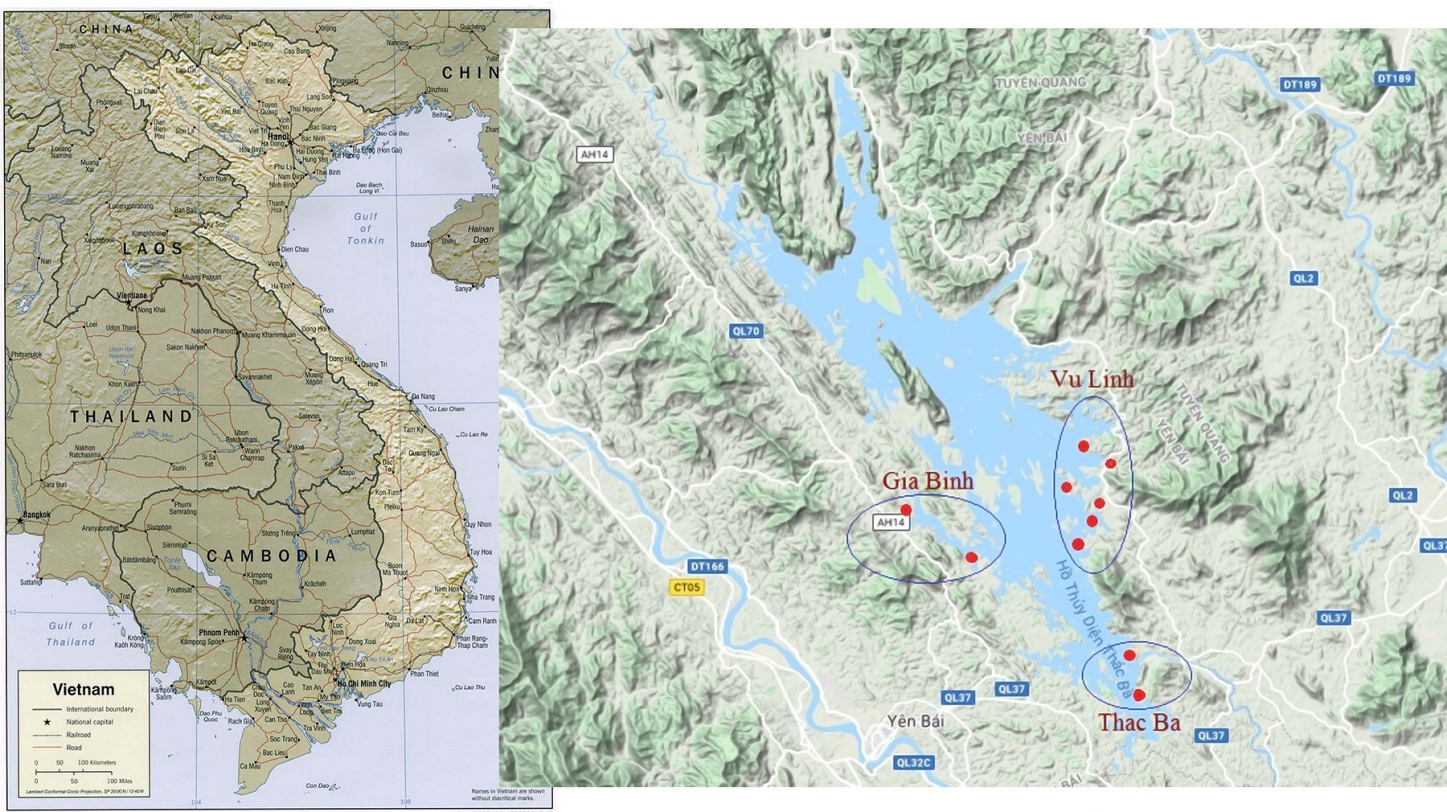
Study sites (red dots) in the Thac Ba reservoir.

Ten sites within the reservoir were selected for snail collection, including 2 sites at Gia Binh, 2 sites at Thac Ba, and 6 sites at Vu Linh commune (Fig 1). Snails were collected every four months from April 2015 to April 2019. During morning hours, snails were collected by hand for 30 minutes per site. Snails were transferred to plastic containers and transported alive to the laboratory where they were identified following the keys of Brandt [31] and Dang [32]. All snails were recorded and checked for trematode infection using the shedding method. Depending on the size of each snail, each individual was placed into a small plastic container with 5 or 10 ml of water each and left for 48 hours to shed cercariae. Snails were checked each day for shedding at 8:00 AM, 1:30 PM and 5:30 PM. Cercariae of *C*. *sinensis* were identified based on their morphological characters [7] and they were taken randomly from each infected snail to confirm their identity using molecular technique (see below). Cercariae of other trematode species were identified to major types according to the keys of Ginetsinskaja [33], Shell [34] and other available references.

### Experimental infection of snails

Five snail species were chosen for experimental infection, including 4 species from Vietnam: *M*. *tuberculata*, *T*. *granifera*, *B*. *fuchsiana*, *P*. *manchouricus* and 2 species from Russia *Koreoleptoxis amurensis* and *P*. *manchouricus*. For experimental infection, snails were collected from trematode-free locations of the suburb of Hanoi, Vietnam and Primorsky Region, Russia. Snails were acclimatized to laboratory conditions for seven days and fed dried lettuce leaves.

For maintenance of experimental snails, aquaria with a bottom layer of sand and small stones were filled (plastic boxes with 2L capacity) with decholorinated tap water. To mimic the natural habitat of these snails, a few pieces of brushwood (*Terminalia catappa*) and aquatic plants (*Hygrophila difformis*, *Lemna minor*) were added to each aquarium. Ten individuals of the same snail species were housed in each aquarium. The number of each snail species used in this experiment varied.

For collecting the *C*. *sinensis* samples from Vietnam, livers and gall bladders of 7 cats were purchased in a slaughter house in Nam Dinh province where restaurants serving cat meat are popular (even if it is technically illegal) [35]. After that, they were placed on ice and carried fresh to the laboratory, the duration for transporting samples were 1.5 hours. In the laboratory, samples were necropsied immediately to find trematodes. The *C*. *sinensis* samples from Russia were collected from experimentally infected rats. A total of 87 adult specimens of *C*. *sinensis* from Vietnam and 15 specimens from Russia, were kept alive in saline at 37 ^o^C for egg release. After 6 hours, the uteri of all *C*. *sinensis* specimens were opened and eggs collected. Eggs were placed in 20 g of feces collected from parasite free domestic cats and 700 ml of tap water was added and shaken until the fecal matter disintegrated. A multichannel pipette (8 channels) with 1 mL tips was used to transfer 5 ml of fecal-egg suspension to each aquarium for experimental infection. When drawing up the suspension, the tip of the pipette reached to the bottom of the glass flask containing the suspension to make sure that eggs are present in the aquaria. After 3 weeks, living snails in each aquarium were recorded and fixed in absolute ethanol for molecular analyses.

### Multiplex PCR for detecting *C*. *sinensis* larvae in snails

Snail specimens were individually crushed. Shell fragments and the muscular foot of the snail were removed, and only the hepatopancreas, which was easily freed from the remaining soft tissue, was kept for extracting genomic DNA. The DNA samples were extracted using the HotSHOT technique [36]. The ITS1 rDNA region of *C*. *sinensis* and the 28S rDNA gene of the snails were co-amplified using two sets of primers designed using the Oligo Calc. program (http://biotools.nubic.northwestern.edu/OligoCalc.html), including: CSF (5'−TGTTCTACATGTATGTTCCGC−3’, forward), CSR (5'−TAGCTCGAGACACATCATGC−3’, reverse), for ITS region; 28SF (5'−AGTAACGGCGAGTGAAGCG−3’, forward), 28SR (5−CTACCGAGCTGAATTCCGC−3’, reverse) for 28S region. The PCR reaction mixture (20μl of total volume) contained 1μl (5 pM) of each primer, 10 μl of Hot Start Green-Taq Master Mix (2 ×) (Promega Corporation, USA) and approximately 10 ng of total DNA. PCR was performed on an Eppendorf Mastercycler using the following cycling conditions: a 5 min initial denaturation step at 95°C, 38 cycles of 25 sec at 95°C, 25 sec at 57°C and 35 sec at 72°C, and a 5 min extension at 72°C. The PCR products were separated by electrophoresis using a 2% agarose gel stained with RedSafe (cat # 21141, iNtRON).

Negative controls (PCR mix with H_2_O instead of DNA) were included, along with positive controls (20 ng DNA of *C*. *sinensis* and 20 ng DNA of non-infected snails). The optimal concentration of DNA for both the snail and *C*. *sinensis* was evaluated using two different concentrations: 10 ng of snail DNA and 100 ng of *C*. *sinensis* DNA; 100 ng of snail DNA and 10 ng of *C*. *sinensis* DNA.

PCR products were purified by ExosapIT enzymatic cleanup and sequenced using Sanger sequencing method. The four primers CSF, CSR, 28SF and 28 SR mentioned above were used for sequencing.

## Results

### Natural trematode infections

A total of 11,985 snails, representing 10 species, were collected (Table 2). Among them, 11,237 snails were examined for cercarial shedding. Five snail species, including *M*. *tuberculata*, *T*. *granifera* (Thiaridae), *P*. *manchouricus*, *B*. *fuchsiana* (Bithyniidae) and *L*. *swinhoei* (Lymnaeidae), were found shedding cercariae. The prevalence of infection in snails ranged from 0.7% to 11.5%.

**Table 2:** Prevalence of infected snails from Thac Ba reservoir.

Snail species	Examined for actively shedding		Examined for *C*. *sinensis* by PCR	
	No. snails examined	No. infected snails (%)	No. snails examined	No. snails infected (%)
**Thiaridae**				
*Melanoides tuberculata*	2,013	151 (7.5)	265	0
*Tarebia granifera*	288	33 (11.5)	116	0
*Thiara scabra*	85	0	−	−
**Viviparidae**				
*Angulyagra polyzonata*	3,386	0	−	−
*Sinotaia aeruginosa*	675	0	−	−
**Lymnaeidae**				
*Lymnaea swinhoei*	426	3 (0.7)	−	−
**Ampullariidae**				
*Pomacea canaliculata*	107	0	−	−
**Bithyniidae**				
*Parafossarulus manchouricus*	2,376	131 (5.5)	120	5 (4.2)
*Bithynia fuchsiana*	1,663	103 (6.2)	120	0
*Gabbia longicornis*	318	0	−	−
**Total**	11,237	421	721	5

A total of seven cercariae types were recorded. Cercariae of *C*. *sinensis* belong to the pleurolophocercous group and can be identified from others within the group by distinct morphological characteristics and their overall movement. In Table 3, we arranged separately *C*. *sinensis* from other species with pleurolophocercous cercariae. *P*. *manchouricus* released all 7 cercariae types, while the other 4 snail species released from 1 to 5 types of cercariae.

**Table 3:** Cercariae types released from the first intermediate hosts.

Cercariae type	Snail species					No. snails (%)
	*M*. *tuberculata*	*T*. *granifera*	*L*. *swinhoei*	*P*. *manchouricus*	*B*. *fuchsiana*	
Clonorchis sinensis^*^				12		12 (2.85)
Pleurolophocercous	72			59	35	156 (37.05)
Xiphidiocercous	48		1	41	33	123 (29.21)
Echinostome	29	33	2	12	31	107 (25.42)
Gymnocephalous	2			3		5 (1.19)
Furcocercous				5	4	9 (2.14)
Monostome				9		9 (2.14)
Total	151	33	3	131	103	421 (100)

Note

* Also Pleurolophocercous.

The most common cercarial type (37.05%) of all cercariae recorded were pleurolophocercous (excluding those of *C*. *sinensis*), this type was found in *M*. *tuberculata*, *P*. *manchouricus* and *B*. *fuchsiana*. The xiphidiocercous type was the second most common (29.21%) and was released from 4 snail species. All infected snail species released echinostome cercariae which was the third most common (25.42%) type. The cercariae of *C*. *sinensis* composed only 2.85% of the total infections and were found only in *P*. *manchouricus*. Two cercariae types, furcocercous and monostome, each constituted 2.14%. Notably, the monostome type was found only in *P*. *manchouricus* while the furcocercous type was shed from both bithyniid species. The gymnocephalous type was the least common with a percentage of 1.19%.

Four snail species, including *M*. *tuberculata*, *T*. *granifera* (Thiaridae), and *P*. *manchouricus* and *B*. *fuchsiana* (Bithyniidae) were examined using the multiplex PCR approach for detecting *C*. *sinensis* (Table 2). Only *P*. *manchouricus* was found to be infected with *C*. *sinensis* with an overall prevalence of 4.2%. The length of the ITS1-rDNA sequence of *C*. *sinensis* was 271 bp while the sequence of the 28S-rDNA fragment of *P*. *manchouricus* was 397 bp in length. Sequences of *C*. *sinensis* and *P*. *manchouricus* were deposited to the NCBI database under accession numbers MT497281 and MT487283, respectively. Samples of three other snail species, *B*. *fuchsiana*, *M*. *tuberculata*, and *T*. *granifera*, were *C*. *sinensis* PCR negative (primers CSF and CSR) but still amplified snail DNA (primers 28SF and 28SR). The GenBank accession numbers for *B*. *fuchsiana*, *M*. *tuberculata*, and *T*. *granifera* are MT497280, MT497282, and MT497284, respectively.

### Experimental infection of snails by *C*. *sinensis*

A total of 1,450 snails of 5 species were used for experimental infection (Table 4). After 21 days post egg exposure, only *P*. *manchouricus* was found infected. The overall prevalence of infection was 7.87%. Additionally, cross-infected snails using eggs of *C*. *sinensis* from Russia and Vietnam were found. Also, of note, one multiplex PCR sample only amplified *C*. *sinensis* DNA and not snail DNA.

**Table 4:** The snails’ survival, infection after 21 days of exposure to *C*. *sinensis* eggs.

Snail species	Snail source	Eggs of *C*. *sinensis* from naturally infected cats (Vietnam)			Eggs of *C*. *sinensis* from experimentally infected rats (Russia)		
		No. snails exposed	No. snail surviving	No. snails infected (%)	No. snails exposed	No. snail surviving	No. snails infected (%)
**Bithyniidae**							
*B*. *fuchsiana*	Vietnam	310	236	0	90	49	0
*P*. *manchouricus*	Vietnam	200	156	17 (10.9%)	60	44	4 (9.10%)
	Russia	100	77	3 (3.90%)	40	28	0
**Semisulcospiridae**							
*Koreoleptoxis amurensis*	Russia	100	26	0	50	15	0
**Thiaridae**							
*M*. *tuberculata*	Vietnam	400	211	0	50	3	0
*T*. *granifera*	Vietnam	150	118	0			

## Discussion

The presence of mollusk first intermediate hosts is the major limiting factor for the distribution of all digenean species including, *C*. *sinensis*. Species of Bithyniidae, which are listed as the first intermediate host of *C*. *sinensis*, are widely distributed [37]. Likewise, the potential second intermediate (fish) and definitive (mammals) hosts are geographically widespread, but *C*. *sinensis* occupies only a limited territory of South and South-East Asia. The northernmost boundary (51−52°N latitude) of *C*. *sinensis* distribution coincides with that of *P*. *manchouricus* [38]. Due to the importance of first intermediate hosts, it is necessary to know which snail species can serve as intermediate hosts in a given area in order to implement measures to control the potential of spread of *C*. *sinensis*. The southernmost border of the distribution of *C*. *sinensis* is in the northern part of Vietnam [1,2,5] although bithyniid species, other than *P*. *manchouricus* are widespread throughout the whole country [39].

The successful experimental infection of *P*. *manchouricus* with *C*. *sinensis*, as well as the finding of cercariae of *C*. *sinensis* in this snail species in Thac Ba reservoir confirm that *P*. *manchouricus* is the most important first intermediate host of the Chinese liver fluke. In this study, the experimental infection rate (7.87%) was lower than that (12.5%) reported by Liang et al. [6]. The difference may be due to differences in the experimental conditions such as snail density, time of maintenance of snails in aquaria before experimental exposure, the maturation of miracidia in eggs, experimental duration, etc. *P*. *manchouricus* is a host to numerous digenean species, and experimental infections have shown it to host at least 5 trematode species in Hanoi, Vietnam, i.e. *Cyathocotyle orientalis* (furcocercous), *Echinochasmus beleocephalus* (echinostome), *Notocotylus intestinalis* (monostome), *Gymnocephala* sp. (gymnocephalous), and *Prosthogonimus* sp. (xiphidiocercous) [40]. Besprozvannykh et al. [28] also found 5 cercariae types from *P*. *manchouricus* in North Vietnam, namely *Echinochasmus japonicus* (echinostome), *N*. *intestinalis* (monostome), *Sphaeridiotrema monorchis* (gymnocephalous), *Holostephanus* sp. (furcocercous), Pleurogenidae gen. sp. (xiphidiocercous). This study only identified cercariae to major groups, but at least 7 trematode species use *P*. *manchouricus* as a first intermediate host. Thus, the diversity of digenean within *P*. *manchouricus* in this area should be studied further.

In addition, for *C*. *sinensis*, a cross-infection of snails from different countries (Vietnam and Russia) was obtained in this experiment. In spite of the fact that statistically significant differences were not found between parasites from Russia and Vietnam using both mitochondrial and nuclear markers [41,42], adaptations and some peculiarities are formed due to the geographic remoteness of the regions, including at the host-parasite level. Worms interacting with the hosts on a common territory are less pathogenic for them, while mollusks from another region might not have developed protective mechanisms. Thus, accidental transfer of the parasite to other regions may contribute to its rapid uncontrolled spread, since the first intermediate hosts will be more sensitive to infection.

Other bithyniid snail species such as *B*. *fuchsiana*, which has been confirmed as a first intermediate host of *C*. *sinensis* in China by the LAMP technique [8], was found uninfected with *C*. *sinensis* in this study. Although the LAMP technique is quite sensitive, Chen et al. [8] did not use DNA sequencing to verify their results, and only indicated that they “are able to confirm the correct products by sequencing”. Moreover, as a control, they used only one representative of the family Opisthorchiidae, *Opisthorchis viverrini*, which usually does not co-occur with *C*. *sinensis* [1,2]. It is possible that these primers may be sensitive to other parasites occurring in the same area, for example, representatives of the genus *Metorchis*. Chung [43] also failed to infect *Bithynia misella* and *B*. *tentaculata* with *C*. *sinensis* experimentally and suggested that both species do not need to be emphasized in snail survey for clonorchiasis even though *B*. *misella* was reported as an intermediate host of *C*. *sinensis* in China [13]. The prevalence of infection of *B*. *fuchsiana* by trematode larvae in Thac Ba reservoir was higher than that recorded for *P*. *manchouricus* (6.2% vs. 5.5%). However, the prevalence of infections by cercariae of *C*. *sinensis* was contrasting, 9.16% (12/131) in *P*. *manchouricus* and 0% in *B*. *fuchsiana*. Multiplex PCR also did not detect *C*. *sinensis* from 120 specimens of *B*. *fuchsiana*, which suggests that the prevalence of infection of *B*. *fuchsiana* in the wild is very low or *B*. *fuchsiana* is not a suitable host of *C*. *sinensis* in this region.

In Vietnam, the first report of *M*. *tuberculata* as the first intermediate host of *C*. *sinensis* was published by Kino et al. [20], and the prevalence of infection was 13.3% in Ninh Binh province. De [21] reported a prevalence of *C*. *sinensis* infections in *M*. *tuberculata* of 10.2% in the Red River Delta. Bui et al. [26] did not find any cercariae of *C*. *sinensis* from 3,335 specimens of *M*. *tuberculata* and 34 specimens of *T*. *granifera* in Nam Dinh province while prevalence of infection with small intestinal trematode larvae was high. Similarly, Nguyen et al. [27] also did not find any *C*. *sinensis* cercariae from 758 specimens of *M*. *tuberculata* in Ninh Binh province which is the most important endemic area for clonorchiasis in Vietnam [21]. The contrasting results may be based on misidentification of cercariae. Cercariae of *C*. *sinensis* belong to “pleurolophocercous” type. This type is found within three families, i. e. Opisthorchiidae, Cryptogonimidae, and Heterophyidae [44,45]. Bui et al. [26] found that parapleurolophocercous (cercariae of the subfamily Haplorchiinae) constituted 40.3% of all infections found in snails while pleurolophocerca only constituted 0.3% in Nam Dinh province, an endemic area of *C*. *sinensis*. Pinto [45] suggested that of the term “parapleurolophocercous” should no longer be used and these cercariae should be referred to as pleurolophocercous. Moreover, if *M*. *tuberculata* was the first intermediate host of *C*. *sinensis*, it would be difficult to explain why *C*. *sinensis* only occurs in northern Vietnam while its intermediate host, *M*. *tuberculata*, is distributed throughout the entire country. The failure to infect *M*. *tuberculata* and *T*. *granifera* by *C*. *sinensis* in this study suggests that these two snail species should be removed from the list of first intermediate hosts of *C*. *sinensis*.

*Koreoleptoxis amurensis* is distributed throughout the endemic areas of *C*. *sinensis* in Russia [7,46]. This snail species has not been reported to be infected with *C*. *sinensis*, however it has been found shedding cercariae of many trematode species, e.g. *Nanophyetus salmincola* (Nanophyetidae), *Plagioporus* sp. (Opecoelidae), *Sanguinicola* sp. (Sanguinicolidae), *Echinochasmus* sp., *Microparyphyim* sp. (Echinostomatidae), *Metagonimus* spp., *Pygidiopsis* sp., and *Centrocestus* (Heterophyidae), and xiphidiocercous cercariae [44]. In addition, this snail belongs to the family Semisulcospiridae, representatives of which were also listed as first intermediate hosts of *C*. *sinensis* in China [14,22]. The failure to infect *K*. *amurensis* by *C*. *sinensis* in this study confirms that this snail species is not a suitable host for *C*. *sinensis*.

The multiplex PCR approach has been used for the detection or the differential diagnosis of *C*. *sinensis* and other trematodes [47,48]. This method, however, has not been reported to detect *C*. *sinensis* DNA from within its host snail. The present study provides a novel multiplex PCR for amplification of multiple genes of both snail and *C*. *sinensis* DNA. This is the first report of using a multiplex PCR approach to detect larvae of *C*. *sinensis* within their snail first intermediate host. Prevalence of infection by *C*. *sinensis* within *P*. *manchouricus* in Thac Ba reservoir, detected by multiplex PCR, was 4.2%, which is nearly 1.5 times higher than the prevalence detected by observation for cercariae shedding (2.85%).

The results obtained can be used to clarify the list of the first intermediate hosts for *C*. *sinensis* throughout its range and show that in the case of studying any aspect related to trematodes, it is necessary to correctly identify not only the parasite species but also their hosts. Since wrong data incorrectly indicate the epidemiological potential of any parasite, which can lead to sudden unpredictable outbreaks of diseases in the regions. In addition, other situations possible when the spread of trematodes may be expected in the region due to erroneous identification of the parasite. For example, parasites that were previously recorded in the mollusks *M*. *tuberculata* and *T*. *granifera*, not being *C*. *sinensis*, in the presence of all conditions for life cycle, can continue to spread in the region without control until they reach a critical threshold. In both cases, it affects the wrong investment in the development of preventive, diagnostic and therapeutic measures that contribute to the health of the population.

In conclusion, we have shown, through both examination of naturally infected snails, as well as experimental infections, that in Northern Vietnam the two thiarids, *M*. *tuberculata* and *T*. *granifera* are not suitable first intermediate hosts of *C*. *sinensis*. Other snail species, which were reported as the first intermediate host of *C*. *sinensis*, should be reconfirmed by molecular data and/or experimental infection.

## References

[1] Mas-Coma S, Bargues MD. Human liver flukes: a review. Research and Reviews in Parasitology. 1997;57:145–218.

[2] Nguyen MH, Madsen H, Fried B. Global status of fish-borne zoonotic trematodiasis in humans. Acta Parasitologica. 2013;58:231–258. 10.2478/s11686-013-0155-5 23990419

[3] Rim HJ, Farag HF, Sornmani S, Cross JH. Food-borne Trematodes: Ignored or Emerging? Parasitology Today. 1994;10:207–209.

[4] Markell EK, Goldsmith R. Trematode infections exclusive of Schistosomiasis. Diseases caused by liver flukes: Clonorchiasis. In: GT Strickland (ed.) Hunter’s Tropical Medicine. 6th Edition, Philadelphia, Saunders 1984;740–755.

[5] Qian MB, Chen YD, Liang S, Yang GJ, Zhou XN. The global epidemiology of clonorchiasis and its relation with cholangiocarcinoma. Infectious Diseases of Poverty. 2012;1:4 10.1186/2049-9957-1-4 23849183 PMC3710150

[6] Liang C, Hu XC, Lv ZY, Wu ZD. Experimental establishment of life cycle of *Clonorchis sinensis*. Chinese Journal of Parasitology and Parasitic Diseases. 2009;27:148–150. (In Chinese). 19856506

[7] Besprozvannykh VV, Ermolenko AV, Rumiantseva EE, Voronok VM, Bartkova AD. *Clonorchis sinensis* and Clonorchiasis in the Primorye Territory. Dalnauka Press, Vladivostok; 2013:82 p. (In Russian). 10.14411/fp.2013.017

[8] Chen Y, Wen T, Lai DH, Wen YZ, Wu ZD, Yang TB, et al Development and evaluation of loop-mediated isothermal amplification (LAMP) for rapid detection of *Clonorchis sinensis* from its first intermediate hosts, freshwater snails. Parasitology. 2013;140:1377–1383. 10.1017/S0031182013000498 23870065

[9] Gibson JB, Sun T. Clonorchiasis In: Marcial-Rojas RA (ed.) Pathology of protozoal and helminthic diseases with clinical correlation. Baltimore, Williams & Wilkins 1971;546–566.

[10] Rim HJ. Clonorchiasis: an update. Journal of Helminthology. 1995;79:269–281. 10.1079/joh2005300 16153321

[11] WHO. Control of foodborne trematode infections. Report of a WHO Study Group. WHO Technical Report Series. 1995;849:1–157. 7740791

[12] Zhang R, Gao S, Geng Y, Huang D, Yu L, Zhang S, et al Epidemiological study on *Clonorchis sinensis* infection in Shenzhen area of Zhujiang delta in China. Parasitology Research. 2007;101:179–183. 10.1007/s00436-006-0441-3 17216484

[13] Guo YH, Wang CM, Luo J, He HX. Intermediate host of main parasites: molluscs distributed in Beijing region. Chinese Journal of Vector Biology and Control. 2009;20:449–453. (In Chinese).

[14] Lu XT, Gu QY, Limpanont Y, Song LG, Wu ZD, Okanurak K, et al Snail-borne parasitic diseases: and update on lobal epidemiological distribution, transmission interruption and control methods. Infectious Diseases of Poverty. 2018;7:28 10.1186/s40249-018-0414-7 29628017 PMC5890347

[15] Tran VQ, Nguyen VT, Nguyen THY, Nguyen THC, Nguyen VP, Hoang MD. Study on some biological and pathological features of *Clonorchis sinensis* infection. Journal of Science and Development. 2012;10:444–450. (In Vietnamese).

[16] Doanh PN, Nawa Y. *Clonorchis sinensis* and *Opisthorchis* spp. in Vietnam: current status and prospects. Transactions of the Royal Society of Tropical Medicine and Hygiene. 2016;110:13–20. 10.1093/trstmh/trv103 26740358

[17] Lu YX, Yang LD, Hu M, Gui AF, Zuo SL. *Parafossarulus anomalospiralis*: first intermediate host of *Clonorchis sinensis*: a first report in Hubei Province, China. Chinese Journal of Parasitology and Parasitic Diseases. 1994;12: 290. (In Chinese).

[18] Yoshida Y. Clonorchiasis- a historical review of contributions of Japanese parasitologists. Parasitology International. 2012;61:5–9. 10.1016/j.parint.2011.06.003 21749930

[19] Chai JY, Murrell KD, Lymbery AJ. Fish-borne parasitic zoonoses: status and issues. International Journal for Parasitology. 2005;35:1233–1254. 10.1016/j.ijpara.2005.07.013 16143336

[20] Kino H, Inaba H, Nguyen VD, Le VC, Dang TS, Hoan TH, et al Epidemiology of clonorchiasis in Ninh Binh province, Vietnam. The Southeast Asian Journal of Tropical Medicine and Public Health. 1998;29:250–254. 9886107

[21] De NV. Fish-borne trematodes in Vietnam. The Southeast Asian Journal of Tropical Medicine and Public Health. 2004;35:229–231.

[22] Liu YY, Zhang WZ, Wang YX. First intermediate hosts of lung fluke in China. Chinese Journal of Zoology. 1984;2:1–5. (In Chinese).

[23] Lun ZR, Gasser RB, Lai DH, Li AX, Zhu XQ, Yu XB, et al Clonorchiasis: a key foodborne zoonosis in China. The Lancet Infectious Diseases. 2005;5:31–41. 10.1016/S1473-3099(04)01252-6 15620559

[24] Madsen H, Frandsen F. The spread of freshwater snails including those of medical and veterinary importance. Acta Tropica. 1989;46:139–146. 10.1016/0001-706x(89)90030-2 2566266

[25] Madsen H., Nguyen MH. An overview of freshwater snails in Asia with main focus on Vietnam. Acta Tropica. 2014;140:105–117. 10.1016/j.actatropica.2014.08.005 25149356

[26] Bui TD, Madsen H, Dang TT. Distribution of freshwater snails in family-based VAC ponds and associated water bodies with special reference to intermediate hosts of fish-borne zoonotic trematodes in Nam Dinh province, Vietnam. Acta Tropica. 2010;116:15–23. 10.1016/j.actatropica.2010.04.016 20457118

[27] Nguyen MH, Do TD, Nguyen TLA, Phan TV, Bui NT, Nguyen VH, et al Current status of fish-borne zoonotic trematode infections in Gia Vien district, Ninh Binh province, Vietnam. Parasites & Vectors. 2015;8:21. 25586313 10.1186/s13071-015-0643-6 PMC4299372

[28] Besprozvannykh VV, Ermolenko AV, Ngo HD, Ha NV, Hung NM, Rozhkovan KV Descriptions of digenean parasites from three snail species, *Bithynia fuchsiana* (Morelet), *Parafossarulus striatulus* Benson and *Melanoides tuberculata* Müller, in North Vietnam. Helmintologia. 2013;50:190–204.

[29] Bui NT, Pham TT, Nguyen TN, Nguyen VH, Murrell D, Phan TV. The importance of wild fish in the epidemiology of *Clonorchis sinensis* in Vietnam. Parasitology Research. 2016;115:3401–3408. 10.1007/s00436-016-5100-8 27160330

[30] Phan TV, Bui NT, Nguyen VH, Murrell D. Comparative risk of liver and intestinal fluke infection from either wild-caught or culture fish in Vietnam. Vector-Borne and Zoonotic Diseases. 2016;16:790–796. 10.1089/vbz.2016.1997 27828767

[31] Brandt RAM (1974) The non-marine aquatic mollusca of Thailand. Archiv für Molluskenkunde 105: 1–423.

[32] Dang NT (1980) Identification of freshwater invertebrates in Northern Vietnam. Hanoi, Science and Technical Press 440–490. (In Vietnamese).

[33] Ginetsinskaya TA. Trematodes, their life cycles, biology and evolution. NewDelhi: Amerind Publishing Company (Translation of the original Russian edition, 1968); 1988.

[34] Schell SC. Handbook of Trematodes of North America (North of Mexico). Idaho: University Press of Idaho; 1985.

[35] Nguyen MH, Tatonova YV, Madsen H. Infections by hepatic trematodes in cats from slaughterhouses in Vietnam. Journal of Parasitology. 2018;104:306–309. 10.1645/18-5 29466091

[36] Truett GE, Heeger P, Mynatt AA, Walker JA, Warman ML. Preparation of PCR-quality mouse genomic DNA with hot sodium hydroxide and tris (HotSHOT). BioTechniques. 2000;29:52–54. 10.2144/00291bm09 10907076

[37] Lydeard C, Cummings KS. Freshwater Mollusks of the World: A Distribution Atlas. JHU Press; 2019.

[38] Prozorova LA, Rasshepkina AV. Reproductive morphology of the genus *Parafossarulus* Annandale, 1924 (Caenogastropoda: Rissooidea: Bithyniidae) with comments on its taxonomy and distribution. The Bulletin of the Russian Far East Malacological Society. 2017;21:178–187.

[39] Thanh DN, Hai HN. Freshwater Mollusca: Gastropoda and Bivalvia In: Fauna of Vietnam. Vietnam Science and Technology Publisher; 2010. (In Vietnamese).

[40] Le NT, Doanh PN, The DT. Trematode larvae in the snail Parafossarulus striatulus (Bithyniidae) collected from Ba Vi and Ngoc Tao areas of Ha Tay. Journal of Biology. 2000;22:15–21. (In Vietnamese).

[41] Chelomina GN, Tatonova YV, Hung NM, Ngo HD. Genetic diversity of the Chinese liver fluke Clonorchis sinensis from Russia and Vietnam. International Journal for Parasitology. 2014;44:795–810. 10.1016/j.ijpara.2014.06.009 25123068

[42] Tatonova YV, Chelomina G, Nguyen HM. Inter-individual and intragenomic variations in the ITS region of Clonorchis sinensis (Trematoda: Opisthorchiidae) from Russia and Vietnam. Infection, Genetics and Evolution. 2017;55:350–357. 10.1016/j.meegid.2017.10.008 28993292

[43] Chung PR. A. comparative study of three species of Bithyniidae (Mollusca: Prosobranchia): *Parafossarulus manchouricus*, *Gabbia misella* and *Bithynia tentaculata*. Malacology Review. 1984;17:1–66.

[44] Sewell RBS. Cercariae indicae. Indian Journal of Medical Research. 1922;10:1–370.

[45] Pinto HA. Pleurolophocercous and parapleurolophocercous types of cercariae: Revisiting concepts. Parasitology International. 2019;68:92–94. 10.1016/j.parint.2018.09.001 30195061

[46] Tatonova YV, Solodovnik DA, Nguyen MH. Human parasites in the Amur River: the results of 2017–2018 field studies. Regional Problems. 2018;3:34–36.

[47] Le TH, Nguyen VD, Blair D, Sithithaworn P, McManus D. *Clonorchis sinensis* and *Opisthorchis viverrini*: Development of a mitochondrial-based multiplex PCR for their identification and discrimination. Experimental Parasitology. 2006;112:109–114. 10.1016/j.exppara.2005.09.012 16310774

[48] Kaewkong W, Intapan PM, Sanpool O, Janwan P, Thanchomnang T, Laumaunwai P, et al Molecular differentiation of Opisthorchis viverrini and Clonorchis sinensis eggs by multiplex real-time PCR with high resolution melting analysis. The Korean Journal of Parasitology. 2013;51:689–694. 10.3347/kjp.2013.51.6.689 24516275 PMC3916459

