# Supplementary material for: Are Melanoides tuberculata and Tarebia granifera (Gastropoda, Thiaridae), suitable first intermediate hosts of Clonorchis sinensis in Vietnam?
Source: PLoS Negl Trop Dis. 2021 Jan 25;15(1):e0009093. doi: 10.1371/journal.pntd.0009093 (PMC7872296; doi:10.1371/journal.pntd.0009093)
Supplement: Attachment — Submitted filename: Verify the role of thiarid snails_2020_PLOS NTD_REVISED.doc [file pntd.0009093.s001.doc]

**Are the thiarids, *Melanoides tuberculata* and *Tarebia granifera*, first intermediate hosts for *Clonorchis sinensis in Vientnam*?**

Running title: Identifying the first intermediate host of *C. sinensis* ***in Vientnam***

**Hung Manh Nguyen^1,4^ *, Hien Hoang Van^1^, Loan Thi Ho^1^, Yulia V. Tatonova^2^, Henry Madsen^3^**

**^1^** Institute of Ecology and Biological Resources (IEBR), Vietnam Academy of Science and Technology (VAST), 18 Hoang Quoc Viet st., Cau Giay dist., Hanoi, Vietnam

**^2^** Federal Scientific Center of the East Asia Terrestrial Biodiversity, Far Eastern Branch, Russian Academy of Sciences, 159 100-letiya st., Vadivostok 690022, Russia

**^3^** Parasitology and Aquatic Diseases, Department of Veterinary Disease Biology, Faculty of Health and Medical Sciences, University of Copenhagen, Thorvaldsensvej 57, 1871 Frederiksberg C, Copenhagen, Denmark

**^4^** Graduate University of Science and Technology, Vietnam Academy of Science and Technology (VAST), 18 Hoang Quoc Viet st., Cau Giay dist., Hanoi, Vietnam

Corresponding should be send to: [*hung_iebr@yahoo.com*](mailto:hung_iebr@yahoo.com) or [*nmhung@iebr.ac.vn*](mailto:nmhung@iebr.ac.vn); telephone number: +84 988 688 203; fax: +844 3836 1196.

**Abstract**

***Background***: Two thiarid species, *Melanoides tuberculata* and *Tarebia granifera* have been reported as the first intermediate hosts of the liver fluke *Clonorchis sinensis*. Their role as intermediate hosts, however, has not been confirmed and is questioned by some researchers?. Here we present studies aimed to clarify the role of these two snail species, by firstly identifying trematodes (cercariae) using molecular technique released from *M. tuberculata* and *T. granifera* and other freshwater snails in a new highly endemic area for *C. sinensis* in Vietnam, the Thac Ba reservoir and secondly an attempt to experimentally infect these snails by eggs of *C. sinensis*.

***Methodology/Principal Findings***: A total of 11,985 snails, representing 10 species were sampled. Five snail species were shedding cercariae with prevalences of infection from 0.7% to 11.5%. Seven cercariae types were recorded. Cercariae of *C. sinensis* were only found in *Parafossarulus manchouricus*. Using the multiplex PCR approach for detecting *C. sinensis* infection, the prevalence in *P. manchouricus* was 4.2%. The accession numbers for *C. sinensis* and the four snail species, *B. fuchsiana*, *P. manchouricus, M. tuberculata* and *T. granifera*, in the NCBI database are MT497281, MT497280, MT497283, MT497282, MT497284, respectively. Only *P. manchouricus* among the 5 snail species exposed was successfully infected with *C. sinensis*, with an infection rate of 7.87%.

***Conclusions/Significance:***

We confirmed that in Vietnam, the two thiarids, *Melanoides tuberculata* and *Tarebia granifera* are not the first intermediate host of *C. sinensis*. Only *P. manchouricus* was found infected by *C. sinensis* in the natural transmission sites, and was the only species that became infected experimentally.

**Keywords**: small liver fluke; freshwater snails; *Parafossarulus manchouricus*; Vietnam

**Authors’ summary**

Up to the present, 13 snail species are reported as first intermediate host for *C. sinensis*, including two species of the Thiaridae, *Melanoides tuberculata* and *Tarebia granifera*. Both snail species have a wide distribution in the World but *C. sinensis* only endemic in East Asia. Their role as hosts, however, has been refuted in other studies. Our study was conducted to clarify this issue. Based on the results from field surveys in a newly discovered and highly endemic area for *C. sinensis*, as well as data obtained under experimental conditions, we confirm that in Vietnam, the two thiarid snail species are not the first intermediate host of *C. sinensis*. *C. sinensis* was only detected in *Parafossarulus manchouricus* by observation, molecular analyses and experimental infection.

**Introduction**

One of the most important fish-borne zoonotic trematodes is *Clonorchis sinensis* (Opisthorchiidae), the small liver fluke that parasitizes humans and fish-eating mammals in some Asian countries [1,2]. The species causes serious diseases in humans and animals, e.g. pericholangitis, pyelophlebitis, cholangiohepatitis and multipleabscesses [3,4] and it is widely distributed in Northeast Asia with high number of humans infected or at risk [1,2,4,5].

Thirteen freshwater snail species have been reported as the first intermediate hosts of *C. sinensis* (Table 1) mainly based on the morphological characters of cercariae (the pleurolophocercous type). Only two species, *Alocinma longicornis* and *Parafossarulus manchouricus*, are confirmed intermediate hosts by experimental infection [6,7] and three species, *A. longicornis*, *Bithynia fuchsiana* and *P. manchouricus* are confirmed by molecular data [8]; the remaining ten species are not yet verified.

**Table 1. List of the first intermediate hosts for *C. sinensis***

| SNAIL SPECIES | COUNTRY OR AREA (Source of information) |
| --- | --- |
| **Assimineidae** |  |
| *Assiminaea* *lutea* | China [9,10] |
| **Bithyniidae** |  |
| **, **Alocinma longicornis* (synonym: *Bithynia longicornis*) | China [8,11−14] |
| *B. chaperi* | Vietnam [1,12] |
| ***Bithynia fuchsiana* | China [8,11−14] |
| *~B. misella* | China [11,13,14], Vietnam [15] |
| *B. siamensis* | Vietnam [16] |
| *Parafossarulus anomalosiralis* | China [11,14,17] |
| **, **P. manchouricus* (synonym: *P. striatulus*) | China [8,11,12,14], Japan [1,18], Korea [1,19], Russia Federation [1,2,7], Vietnam [20,21] |
| *P. sinensis* | China [12,14] |
| **Semisulcospiridae** |  |
| *Semisulcospira cancellata* | China [14,22] |
| *S. libertine* | China [11] |
| **Thiaridae** |  |
| *Melanoides tuberculata* | China [23], Vietnam [20,21] |
| *Thiara granifera* | Taiwan [11] |

Note: * Confirmed by experimental infection ** Confirmed by molecular data; ~ failed in an experimental infection

*Melanoides tuberculata* and *T. granifera* have a high invasion capacity and they are now present in North (southern USA, Mexico), Central (Panama, Caribbean region) and South America (Venezuela, Brazil), Europe, Africa, Australia, Pacific islands and Asia [1,24,25]. Mas-Coma & Bargues [1] mentioned that their wide distribution potentially could results in geographical spread of *C. sinensis*. However, the role of thiarid snails as the first intermediate hosts for liver flukes has been questioned by Bui et al. [26] and Nguyen et al. [27] because they did not find cercariae of *C. sinensis* in thousands of specimens of thiarid snails from areas known to be highly endemic for clonorchiasis in Vietnam. In addition, Besprozvannykh et al. [28] conducted experimental infections in fish with all cercaria types from *M. tuberculata* specimens in Northern Vietnam and they did not find any metacercariae of *C. sinensis*. Similarly, in China, Zhang et al. [12] found *C. sinensis* larvae in four snail species, i.e. *P. manchouricus*, *P. sinensis*, *B. fuchsiana* and *A. longicornis*, while *M. tuberculata* was not infected.

The present study aimed to verify the role of two thiarid snails, *M. tuberculata* and *T. granifera*, as the first intermediate hosts of *C. sinensis* by using different approaches i.e. field surveys, experimental infection and molecular detection of *C. sinensis* infection in snails.

**Materials and Methods**

Study area, snail sampling and examination

Thac Ba reservoir covers an area of 23,400 ha of Yen Bai Province in the mountainous region of Northern Vietnam. There are 12 ethnic groups living in communes around the reservoir, and local people usually prepare and eat raw fish from the reservoir [29]. According to recent studies, the Thac Ba reservoir is known as a new highly endemic area for *C. sinensis* [29,30]. In this region, the sewage system of each household is very simple and domestic waste water and latrine flush directly into canals nearby and from there into the reservoir. Domestic animals, e.g. cattle, pigs, ducks, chicken, dogs, cats are free roaming during the day and locked up during the evening and nights. Manure from domestic animals is likely to enter the reservoir. The coexistence of intermediate hosts (snails and fishes) and the habit of eating raw or insufficiently cooked fish explain the high prevalence of fish-borne zoonotic trematodes in this region.

Ten sites in the reservoir were selected for snail collection, including 2 sites at Gia Binh, 2 sites at Thac Ba, and 6 sites at Vu Linh commune (Fig. 1). Snails were collected each four months from April 2015 to April 2019. During morning hours, collection of snails was conducted for 30 minutes per site by hand picking. Snails were transferred to plastic containers and transported alive to the laboratory where they were identified following the keys of Brandt [31] and Dang [32]. All snails were recorded and checked for trematode infection using the shedding method. Depending on the size of snails, each individual was placed in small plastic container with 5 or 10 ml of water each and left for 48 hours to shed cercariae. Snails were checked each day for shedding at 8:00 AM, 1:30 PM and 5:30 PM. Cercariae of *C. sinensis* were identified based on their morphological characters [7] and they were taken randomly from each infected snail to confirm by using molecular technique (see below). Cercariae of other trematode species were identified to major types according to the keys of Ginetsinskaja [33], Shell [34] and other available references.

**Fig 1. Study sites (red dots) in the Thac Ba reservoir (source of map: google.com/maps/)**

Experimental infection for snails

Five snail species were chosen for experimental infection, including 4 species from Vietnam: *M. tuberculata*, *T. granifera*, *B. fuchsiana*, *P. manchouricus* and 2 species from Russia *Koreoleptoxis amurensis* and *P. manchouricus*. For experimental infection, trematodes-free snails were collected from non-endemic areas, transferred to the laboratory and kept for seven days for acclimation. During this period, dried lettuce leaves were provided as food.

For maintenance of experimental snails, aquaria (plastic boxes with 2l capacity) were set up with a bottom layer of sand and small stones. Furthermore, to mimic the natural habitat of snails a few brushwood (*Terminalia catappa*), aquatic plants (*Hygrophila difformis*, *Lemna minor*) and decholorinated tap water were added. In each aquarium, 10 individuals of the same snail species were kept. The number of each snail species used in this experiment is listed in Table 4.

Eighty seven adult specimens of *C. sisnensis* collected from naturally infected cats from Vietnam [35] and 15 specimens collected form experimental infection of rats from Russia, were kept alive in saline at 37 ^o^C for egg release, separately. Regarding to the samples from Russia, euthanasia of laboratory animals was carried out in accordance with the Committee on the Ethics of Animal Experiments of Federal Scientific Center of the East Asia Terrestrial Biodiversity, Russia (Permit Number: 3 of 02.06.2011). After 6 hours, eggs were collected and added to 20 g feces from domestic cat (free from parasites) and then added to 700 ml tape water in a glass flask (volume 1000 ml) which was then shaken to disintegrate the fecal matter. A total of 5 ml of this suspension was added to each aquarium as food for experimental snails.

After 3 weeks, snails alive in each aquarium were recorded and fixed in absolute ethanol for molecular analyses.

Multiplex PCR for detecting *C. sinensis* larvae in snails

Snail specimens were individually crushed. Shell fragments and the muscular foot of the snail were removed, and only the hepatopancreas, which was easily freed from the remaining soft tissue, was kept for extracting genomic DNA. The DNA samples were extracted by using HotSHOT technique [36]. For the specific amplification the ITS1−rDNA region of *C. sinensis*, and co−amplification of the 28S−rDNA gene of snails, two sets primers were designed by using Oligo Calc. program (<http://biotools.nubic.northwestern.edu/OligoCalc.html>), including: CSF (5'−TGTTCTACATGTATGTTCCGC−3’, forward), CSR (5'−TAGCTCGAGACACATCATGC−3’, reverse), for ITS region; 28SF (5'−AGTAACGGCGAGTGAAGCG−3’, forward), 28SR (5−CTACCGAGCTGAATTCCGC−3’, reverse) for 28S region. The reaction mixture (20µl of total volume) contained 1µl (5 pM) of each primer, 10 µl of Hot Start Green-Taq Master Mix (2 ×) (Promega Corporation, USA) and approximately 10 ng of total DNA. PCR was performed on an Eppendorf Mastercycle using the following cycling conditions: 5 min initial denaturation step at 95 ˚C, 38 cycles of 25 sec at 95 ˚C, 25 sec at 57 ˚C and 35 sec at 72 ˚C, and a 5 min extension at 72 ˚C. The PCR products were separated by electrophoresis on 2% agarose gel stained with GelRed (cat # and company).

Negative controls (PCR mix with H_2_O instead of DNA) were included, along with positive controls of PCR mix with 20 ng DNA of *C. sinensis* and 20 ng DNA of non-infected snail species as template. The concentration of DNA of the snail and *C. sinensis* were evaluated by two different mixes: 10 ng DNA of snail and 100 ng DNA of *C. sinensis*; 100 ng DNA of snail and 10 ng DNA of *C. sinensis*.

The PCR products of positive samples from natural infection were purified by ExosapI and were sequenced using Sanger sequencing method. Four primers CSF, CSR, 28SF and 28 SR mentioned above were used for sequencing.

**Results**

Trematode infection in snails from Thac Ba reservoir

A total of 11,985 snails, representing 10 species, were collected (Table 2). Among them, 11,237 snails were examined by shedding for detection of cercariae. Five snail species, including *M. tuberculata*, *T. granifera* (Thiaridae), *P. manchouricus*, *B. fuchsiana* (Bithyniidae) and *L. swinhoei* (Lymnaeidae), were shedding cercariae. The prevalence of infection in snails ranged from 0.7% to 11.5%.

Table 2. Prevalence of infected snails from Thac Ba reservoir

| **Snail species** | **Examined by shedding** | | **Examined by PCR for detecting *C. sinensis*** | |
| --- | --- | --- | --- | --- |
|  | **No. of snails examined** | **No. of infected snails (%)** | **No. of snails examined** | **No. of snails infected (%)** |
| **Thiaridae** |  |  |  |  |
| *Melanoides tuberculata* | 2,013 | 151 (7.5) | 265 | 0 |
| *Tarebia granifera* | 288 | 33 (11.5) | 116 | 0 |
| *Thiara scabra* | 85 | 0 | − | − |
| **Viviparidae** |  |  |  |  |
| *Angulyagra polyzonata* | 3,386 | 0 | − | − |
| *Sinotaia aeruginosa* | 675 | 0 | − | − |
| **Lymnaeidae** |  |  |  |  |
| *Lymnaea swinhoei* | 426 | 3 (0.7) | − | − |
| **Ampullariidae** |  |  |  |  |
| *Pomacea canaliculata* | 107 | 0 | − | − |
| **Bithyniidae** |  |  |  |  |
| *Parafossarulus manchuricus* | 2,376 | 131 (5.5) | 120 | 5 (4.2) |
| *Bithynia fuchsiana* | 1,663 | 103 (6.2) | 120 | 0 |
| *Gabbia longicornis* | 318 | 0 | − | − |
| **Total** | 11,237 | 421 | 721 | 5 |

Seven cercariae types were recorded (Table 3). *P. manchouricus* released all 7 cercariae types, while the other 4 snail species released from 1 to 5 types of cercariae.

The most common type (37.05%) of all cercariae recorded were pleurolophocercous (excluding cercariae of *C. sinensis*), this cercarial type was found in *M. tuberculata*, *P. manchouricus* and *B. fuchsiana*. The xiphidiocercous was the second most common type (29.21%), and was released from 4 snail species. All infected snail species released echinostome cercariae, and this type was the third most common (25.42%). The cercariae of *C. sinensis* composed only 2.85% of cercariae from infected snails. These cercariae were found only in *P. manchouricus*. Two cercariae types, furcocercous and monostome, each constituted 2.14% of all recorded cercariae. Notably, monostome type was found only in *P. manchouricus* while furcocercous was shed from both bithyniid species. The gymnocephalous was the least common with a percentage of 1.19%.

**Table 3. Cercaria types released from the first intermediate hosts**

| Cercaria type | Snail species | | | | | No. snails (%) |
| --- | --- | --- | --- | --- | --- | --- |
|  | *M. tuberculata* | *T. granifera* | *L. swinhoei* | *P. manchouricus* | *B. fuchsiana* |  |
| *Clonorchis sinensis** |  |  |  | 12 |  | 12 (2.85) |
| Pleurolophocercous | 72 |  |  | 59 | 35 | 156 (37.05) |
| Xiphidiocercous | 48 |  | 1 | 41 | 33 | 123 (29.21) |
| Echinostome | 29 | 33 | 2 | 12 | 31 | 107 (25.42) |
| Gymnocephalous | 2 |  |  | 3 |  | 5 (1.19) |
| Furcocercous |  |  |  | 5 | 4 | 9 (2.14) |
| Monostome |  |  |  | 9 |  | 9 (2.14) |
| Total | 151 | 33 | 3 | 131 | 103 | 421 (100) |

*) Also Pleurolophocercous

Four snail species, including *M. tuberculata*, *T. granifera* (Thiaridae), and *P. manchouricus* and *B. fuchsiana* (Bithyniidae) were examined by the multiplex PCR approach for detecting *C. sinensis* (table 2). Only *P. manchouricus* was found infected with *C. sinensis* with an overall prevalence of 4.2%. The length of the ITS1-rDNA sequence of *C. sinensis* was 271 bp while the sequence of the 28S-rDNA fragment of *P. manchouricus* was 397 bp in length. The accession numbers in the NCBI database of *C. sinensis* and *P. manchouricus* are MT497281 and MT487283, respectively. Samples of three other snail species, *B. fuchsiana*, *M. tuberculata*, and *T. granifera*, were negative with the specific primers CSF and CSR, but positive with primer set 28SF and 28SR. The accession numbers in the NCBI database of *B. fuchsiana*, *M. tuberculata*, and *T. granifera* are MT497280, MT497282, and MT497284, respectively.

Experimental infection of snails by *C. sinensis*

A total of 1,450 snails of 5 species were used in the experimental infection. After 21 days post− infection, only *P. manchouricus* was found infected. The overall prevalence of infection was 7.87%. The cross-infected snails using eggs from Russia and Vietnam were found, noticeably. In addition, one sample has PCR for *C. sinensis* while the PCR of snail was absent.

**Table 4. Number of survived snails and their infection rates at 21 days post−**infection

| Snail species | Snail’s source | Eggs of *C. sinensis* from naturally infected cats (Vietnam) | | | Eggs of *C. sinensis* from experimentally infected rats (Russia) | | |
| --- | --- | --- | --- | --- | --- | --- | --- |
|  |  | **No. of snails exposed** | **No. snail surviving** | **No. snails infected (%)** | **No. of snails exposed** | **No. snail surviving** | **No. snails infected (%)** |
| **Bithyniidae** | | | | | | | |
| *P. manchouricus* | Vietnam | 200 | 156 | 17 (10.9%) | 60 | 44 | 4 (9.10%) |
|  | Russia | 100 | 77 | 3 (3.90%) | 40 | 28 | 0 |
| *B. fuchchsiana* | Vietnam | 310 | 236 | 0 | 90 | 49 | 0 |
| **Thiaridae** | | | | | | | |
| *M. tuberculata* | Vietnam | 400 | 211 | 0 | 50 | 3 | 0 |
| *T. granifera* | Vietnam | 150 | 118 | 0 |  |  |  |
| **Semisulcospiridae** | | | | | | | |
| *Koreoleptoxis amurensis* | Russia | 100 | 26 | 0 | 50 | 15 | 0 |

**Discussion**

Presence of first intermediate host is a limiting factor for distribution of all trematode species including *C. sinensis*. Species of the Bithyniidae, which are listed as first intermediate host of *C. sinensis*, are widely distributed [37]. Likewise, the potential second intermediate and definitive hosts are also present over a wide area, but *C. sinensis* occupies only limited territory of South and South-East Asia. The northernmost boundary (51-52°N latitude) of *C. sinensis* coincides with that of *P. manchouricus* [38]. Due to the importance of the first intermediate hosts, it is necessary to know which snail species can serve as intermediate hosts in a given area in order to implement control measures including snail control against the disease and to assess the potential of spread of *C. sinensis*. The southernmost border of the distribution of *C. sinensis* is in the northern Vietnam [1,2,5] albeit bithyniid species, other than *P. manchouricus* are widespread in the whole country [39].

The successful experimental infection of *P. manchouricus* with *C. sinensis*, as well as the finding of cercariae of *C. sinensis* in this snail species in Thac Ba reservoir confirm that *P. manchouricus* is the most important first intermediate host of the Chinese liver fluke. In this study, the experimental infection rate (7.87%) was lower than that (12.5%) reported by Liang et al. [6]. The difference may be due to differences in the experimental conditions such as snail density, time of maintenance of snails in aquaria before experimental exposure, the maturation of miracidia in eggs, experimental duration, etc. *P. manchouricus* is host of many trematode species. Experimental infections proved it to be host of 5 trematode species in Hanoi suburban, i.e. *Cyathocotyle orientalis* (furcocercous), *Echinochasmus beleocephalus* (echinostome), *Notocotylus intestinalis* (monostome), *Gymnocephala* sp. (gymnocephalous), and *Prosthogonimus* sp. (xiphidiocercous) [40]. Besprozvannykh et al. [28] also found 5 cercariae types from *P. manchouricus* in North Vietnam, namely *Echinochasmus japonicus* (echinostome), *N.s intestinalis* (monostome), *Sphaeridiotrema monorchis* (gymnocephalous), *Holostephanus* sp. (furcocercous), Pleurogenidae gen. sp. (xiphidiocercous). This study only identified cercariae to major groups, but at least 7 trematode species used *P. manchouricus* as intermediate host. Thus, the diversity of trematodes infection in *P. manchouricus* in this area should be studied further.

Other bithyniid snail species such as *B. fuchsiana*, which was confirmed as the first intermediate host of *C. sinensis* in China by LAMP technique [8], did not become infected with *C. sinensis* in this study. Although the method used by the authors is quite sensitive, nevertheless, Chen et al. [8], however, did not use sequencing to verify their results, but only indicated that they “are able to confirm the correct products by sequencing”. Moreover, as a control, they used only one representative of the family Opisthorchiidae, *O. viverrini*, which usually does not co-occur with *C. sinensis* [1,2]. It is possible that these primers may be sensitive to other parasites circulating in the same territory, for example, representatives of the genus *Metorchis*. Chung [41] also failed to infect *B. misella* and *B. tentaculata* with *C. sinensis* experimentally and suggested that both species do not need to be emphasized in snail survey for clonorchiasis even though *B. misella* was reported as intermediate host of *C. sinensis* in China [13]. The prevalence of infection of *B. fuchsiana* by trematode larvae in Thac Ba reservoir was higher than that recorded for *P. manchouricus* (6.2% vs. 5.5%). However, the prevalence of infections by cercariae of *C. sinensis* was contrasting, 9.16% (12/131) in *P. manchouricus* and 0% in *B. fuchsiana*. Multiplex PCR also did not detect *C. sinensis* from 120 specimens of *B. fuchsiana*, which suggests that the prevalence of infection of *B. fuchsiana* in the wild is very low or *B. fuchsiana* is not suitable host of *C. sinensis* in this region.

In Vietnam, the first report of *M. tuberculata* as the first intermediate host of *C. sinensis* was published by Kino et al. [20], and the prevalence of infection was 13.3% in Ninh Binh province. De [21] reported a prevalence of *C. sinensis* infections in *M. tuberculata* of 10.2% in the Red River Delta. Bui et al. [26] did not find any cercariae of *C. sinensis* from 3,335 specimens of *M. tuberculata* and 34 specimens of *T. granifera* in Nam Dinh province while prevalence of infections with small intestinal trematode larvae were high. Similarly, Nguyen et al. [27] also did not find any *C. sinensis* cercariae from 758 specimens of *M. tuberculata* in Ninh Binh province which is the most important endemic area for clonorchiasis in Vietnam [21]. The contrasting results may be based on misidentification of cercariae. Cercariae of *C. sinensis* belong to “pleurolophocercous” type. This type is found within three families, i. e. Opisthorchiidae, Cryptogonimidae, and Heterophyidae [42,43]. Bui et al. [26] found that parapleurolophocercous (cercariae of the subfamily Haplorchiinae) constituted 40.3% of all infections found in snails while pleurolophocerca only constituted 0.3% in Nam Dinh province − an endemic area of *C. sinensis*. Pinto [43] suggested that of the term “parapleurolophocercous” should no longer be used and these cercariae should be refer to as pleurolophocercous as well. Moreover, if *M. tuberculata* is the first intermediate host of *C. sinensis*, it would be difficult to explain why *C. sinensis* only occurs in northern Vietnam while its intermediate host present in whole country. The failure to infect *M. tuberculata* and *T. granifera* by *C. sinensis* in this study suggests that these two snail species should be removed from the list of the first intermediate host of *C. sinensis*.

*Koreoleptoxis amurensis* distributes in the endemic areas of *C. sinensis* in Russia Federation [7,44]. This snail species has not been reported to be infected with *C. sinensis*, however it has been found shedding cercariae of many trematode species, e.g. *Nanophyetus salmincola* (Nanophyetidae), *Plagioporus* sp. (Opecoelidae), *Sanguinicola* sp. (Sanguinicolidae), *Echinochasmus* sp., *Microparyphyim* sp. (Echinostomatidae), *Metagonimus* spp., *Pygidiopsis* sp., and *Centrocestus* (Heterophyidae), and xiphidiocercous cercariae [44]. In addition, this snail belongs to the family Semisulcospiridae, representative of which were also listed as first intermediate hosts of *C. sinensis* in China [14,22]. The failure to infect *K. amurensis* by *C. sinensis* in this study confirms that this snail species is not a suitable host for *C. sinensis*.

The multiplex PCR approach has been used for the detection or the differential diagnosis of *C. sinensis* and other trematodes [45,46]. This method, however, has not been reported to detect the larvae of *C. sinensis* inside its host snails. The present study provides the information of used primers and optimal PCRs for amplification of multiple genes of both snails and *C. sinensis*. This is the first report of using multiplex PCR approach to detect larvae of *C. sinensis* in the host snails. The prevalence of infection by *C. sinensis* from *P. machouricus* in Thac Ba reservoir, which was detected by the multiplex PCR, was 4.2%. This prevalence is nearly 1.5 times higher than the prevalence detected by observation for cercariae shedding (2.85%). The fragments of 28S−rRNA gene of *M. tuberculata*, *T. granifera*, *B. fuchsiana* and *P. manchouricus* from Vietnam have been the first time reported.

In conclusion, when analyzing naturally infected snails, as well as data obtained under experimental conditions, we confirmed that in Vietnam, the two thiarids, *M. tuberculata* and *T. granifera* are not the first intermediate host of *C. sinensis*.

**Acknowledgments**

This research is funded by Vietnam National Foundation for Science and Technology Development (NAFOSTED) under grant number 106.05-2018.17.

**Author Contributions**

Concevied and designed the experiments: HMN HM YVT. Performed the experiments: HMN HVH LTH YVT. Contributed reagents/materials/analysis tools: HMN HVH LTH YVT. Wrote the paper: HMN HM YVT LTH HVH.

**References**

1. Mas-Coma S, Bargues MD (1997) Human liver flukes: a review. Research and Reviews in Parasitology 57: 145–218.

2. Nguyen MH, Madsen H, Fried B (2013) Global status of fish-borne zoonotic trematodiasis in humans. Acta Parasitologica 58: 231–258.

3. Rim HJ, Farag HF, Sornmani S, Cross JH (1994) Food-borne Trematodes: Ignored or Emerging? Parasitology Today 10: 207–209.

4. Markell EK, Goldsmith R (1984) Trematode infections exclusive of Schistosomiasis. Diseases caused by liver flukes: Clonorchiasis. In: Strickland GT (ed.) Hunter’s Tropical Medicine. 6th Edition, Philadelphia, Saunders 740–755.

5. Qian MB, Chen YD, Liang S, Yang GJ, Zhou XN (2012) The global epidemiology of clonorchiasis and its relation with cholangiocarcinoma. Infectious Diseases of Poverty 1: 4

6. Liang C, Hu XC, Lv ZY, Wu ZD (2009) Experimental establishment of life cycle of *Clonorchis sinensis*. Chinese Journal of Parasitology and Parasitic Diseases 27: 148-150. (In Chinese).

7. Besprozvannykh VV, Ermolenko AV, Rumiantseva EE, Voronok VM, Bartkova AD (2013) *Clonorchis sinensis* and Clonorchiasis in the Primorye Territory. Dalnauka Press, Vladivostok, 82 p. (In Russian).

8. Chen Y, Wen T, Lai DH, Wen YZ, Wu ZD, Yang TB, et al. (2013) Development and evaluation of loop-mediated isothermal amplification (LAMP) for rapid detection of *Clonorchis sinensis* from its first intermediate hosts, freshwater snails. Parasitology 140: 1377–1383.

9. Gibson JB, Sun T (1971) Clonorchiasis. In: Marcial-Rojas RA (ed.) Pathology of protozoal and helminthic diseases with clinical correlation. Baltimore, Williams & Wilkins 546–566.

10. Rim HJ (1995) Clonorchiasis: an update. Journal of Helminthology 79: 269–281.

11. WHO (1995) Control of foodborne trematode infections. Report of a WHO Study Group. WHO Technical Report Series 849: 1–157.

12. Zhang R, Gao S, Geng Y, Huang D, Yu L, Zhang S, et al. (2007) Epidemiological study on *Clonorchis sinensis* infection in Shenzhen area of Zhujiang delta in China. Parasitology Research 101: 179–183.

13. Guo YH, Wang CM, Luo J, He HX (2009) Intermediate host of main parasites: molluscs distributed in Beijing region. Chinese Journal of Vector Biology and Control 20: 449–453. (In Chinese).

14. Lu XT, Gu QY, Limpanont Y., Song LG, Wu ZD, Okanurak K, et al. (2018) Snail-borne parasitic diseases: and update on lobal epidemiological distribution, transmission interruption and control methods. Infectious Diseases of Poverty, 7: 28.

15. Tran VQ, Nguyen VT, Nguyen THY, Nguyen THC, Nguyen VP, Hoang MD (2012) Study on some biological and pathological features of *Clonorchis sinensis* infection. Journal of Science and Development 10: 444–450. (In Vietnamese).

16. Doanh PN, Nawa Y (2016). *Clonorchis sinensis* and *Opisthorchis* spp. in Vietnam: current status and prospects. Transactions of the Royal Society of Tropical Medicine and Hygiene 110: 13–20.

17. Lu YX, Yang LD, Hu M, Gui AF, Zuo SL (1994) *Parafossarulus anomalospiralis*: first intermediate host of *Clonorchis sinensis*: a first report in Hubei Province, China. Chinese Journal of Parasitology and Parasitic Diseases 12: 290. (In Chinese).

18. Yoshida Y (2012). Clonorchiasis- a historical review of contributions of Japanese parasitologists. Parasitology International 61: 5–9.

19. Chai JY, Murrell KD, Lymbery AJ (2005) Fish-borne parasitic zoonoses: status and issues. International Journal for Parasitology 35: 1233–1254.

20. Kino H, Inaba H, Nguyen VD, Le VC, Dang TS, Hoan TH, et al. (1998) Epidemiology of clonorchiasis in Ninh Binh province, Vietnam. The Southeast Asian Journal of Tropical Medicine and Public Health 29: 250–254.

21. De NV (2004) Fish-borne trematodes in Vietnam. The Southeast Asian Journal of Tropical Medicine and Public Health 35: 229–231.

22. Liu YY, Zhang WZ, Wang YX (1984) First intermediate hosts of lung fluke in China. Chinese Journal of Zoology 2: 1–5. (In Chinese).

23. Lun ZR, Gasser RB, Lai DH, Li AX, Zhu XQ, Yu XB, et al. (2005) Clonorchiasis: a key foodborne zoonosis in China. The Lancet Infectious Diseases 5: 31–41.

24. Madsen H, Frandsen F (1989) The spread of freshwater snails including those of medical and veterinary importance. Acta Tropica 46: 139–146.

25. Madsen H., Nguyen MH (2014) An overview of freshwater snails in Asia with main focus on Vietnam. Acta Tropica, 140: 105–117.

26. Bui TD, Madsen H, Dang TT (2010) Distribution of freshwater snails in family-based VAC ponds and associated water bodies with special reference to intermediate hosts of fish-borne zoonotic trematodes in Nam Dinh province, Vietnam. Acta Tropica 116: 15–23.

27. Nguyen MH, Do TD, Nguyen TLA, Phan TV, Bui NT, Nguyen VH, et al. (2015) Current status of fish-borne zoonotic trematode infections in Gia Vien district, Ninh Binh province, Vietnam. Parasites & Vectors 8: 21.

28. Besprozvannykh VV, Ermolenko AV, Ngo HD, Ha NV, Hung NM, Rozhkovan KV (2013) Descriptions of digenean parasites from three snail species, *Bithynia fuchsiana* (Morelet), *Parafossarulus striatulus* Benson and *Melanoides tuberculata* Müller, in North Vietnam. Helmintologia 50: 190–204.

29. Bui NT, Pham TT, Nguyen TN, Nguyen VH, Murrell D, Phan TV (2016) The importance of wild fish in the epidemiology of *Clonorchis sinensis* in Vietnam. Parasitology Research 115: 3401–3408.

30. Phan TV, Bui NT, Nguyen VH, Murrell D (2016) Comparative risk of liver and intestinal fluke infection from either wild-caught or culture fish in Vietnam. Vector-Borne and Zoonotic Diseases, 16: 790–796.

31. Brandt RAM (1974) The non-marine aquatic mollusca of Thailand. Archiv für Molluskenkunde 105: 1–423.

32. Dang NT (1980) Identification of freshwater invertebrates in Northern Vietnam. Hanoi, Science and Technical Press 440–490. (In Vietnamese).

33. Ginetsinskaya TA (1988) Trematodes, their life cycles, biology and evolution. NewDelhi: Amerind Publishing Company (Translation of the original Russian edition, 1968).

34. Schell SC (1985) Handbook of Trematodes of North America (North of Mexico). Idaho: University Press of Idaho.

35. Nguyen MH, Tatonova YV, Madsen H (2018) Infections by hepatic trematodes in cats from slaughterhouses in Vietnam. Journal of Parasitology 104: 306–309.

36. Truett GE, Heeger P, Mynatt AA, Walker JA, Warman ML (2000) Preparation of PCR-quality mouse genomic DNA with hot sodium hydroxide and tris (HotSHOT). BioTechniques, 29: 52–54.

37. Lydeard C, Cummings KS (2019) Freshwater Mollusks of the World: A Distribution Atlas. JHU Press.

38. Prozorova LA, Rasshepkina AV (2017) Reproductive morphology of the genus *Parafossarulus* Annandale, 1924 (Caenogastropoda: Rissooidea: Bithyniidae) with comments on its taxonomy and distribution. The Bulletin of the Russian Far East Malacological Society 21: 178–187.

39. Thanh DN, Hai HN (2010) Freshwater Mollusca: Gastropoda and Bivalvia. In: Fauna of Vietnam. Vietnam Science and Technology Publisher (In Vietnamese).

40. Le NT, Doanh PN, The DT (2000) Trematode larvae in the snail Parafossarulus striatulus (Bithyniidae) collected from Ba Vi and Ngoc Tao areas of Ha Tay. Journal of Biology 22: 15–21. (In Vietnamese).

41. Chung PR (1984) A. comparative study of three species of Bithyniidae (Mollusca: Prosobranchia): *Parafossarulus manchouricus*, *Gabbia misella* and *Bithynia tentaculata*. Malacology Review 17: 1–66.

42. Sewell RBS (1922) Cercariae indicae. Indian Journal of Medical Research 10: 1–370.

43. Pinto HA (2019) Pleurolophocercous and parapleurolophocercous types of cercariae: Revisiting concepts. Parasitology International 68: 92–94.

44. Tatonova YV, Solodovnik DA, Nguyen MH (2018) Human parasites in the Amur River: the results of 2017-2018 field studies. Regional Problems 3: 34–36.

45. Le TH, Nguyen VD, Blair D, Sithithaworn P, McManus D (2006) *Clonorchis sinensis* and *Opisthorchis viverrini*: Development of a mitochondrial-based multiplex PCR for their identification and discrimination. Experimental Parasitology 112: 109–114.

46. Kaewkong W, Intapan PM, Sanpool O, Janwan P, Thanchomnang T, Laumaunwai P, et al. (2013) Molecular differentiation of Opisthorchis viverrini and Clonorchis sinensis eggs by multiplex real-time PCR with high resolution melting analysis. The Korean Journal of Parasitology 51: 689–694.
