# Supplementary material for: Are Melanoides tuberculata and Tarebia granifera (Gastropoda, Thiaridae), suitable first intermediate hosts of Clonorchis sinensis in Vietnam?
Source: PLoS Negl Trop Dis. 2021 Jan 25;15(1):e0009093. doi: 10.1371/journal.pntd.0009093 (PMC7872296; doi:10.1371/journal.pntd.0009093)
Supplement: Attachment — Submitted filename: Response to reviewers (PLOS NTD)_5.10.20.docx [file pntd.0009093.s003.docx]

Dear Prof. Xiao-Nong Zhou and Prof. Banchob Sripa,

Thank you very much for reviewing our manuscript “Are the thiarids, *Melanoides tuberculata* and *Tarebia granifera*, first intermediate hosts for *Clonorchis sinensis*” (ref. no. PNTD-D-20-00929). We appreciate the peer-review comments and the opportunity to submit a revised manuscript. We agree with almost all changes suggested by the external reviewers. For the comments, please find our point-by-point response below and also directly in the comment boxes of the revision (with track changes), clearly indicating how and where in the manuscript changes have been made. We used blue text in our response to comments.

We appreciate your kind consideration of our revised manuscript for publication in PLOS NTD and look forward to your decision.

Sincerely,

On behalf of all authors,

Hung Manh Nguyen

Reviewer's Responses to Questions

<b>Key Review Criteria Required for Acceptance?</b></br></br>

As you describe the new analyses required for acceptance, please consider the following:</br></br>

<b>Methods</b></br></br>

-Are the objectives of the study clearly articulated with a clear testable hypothesis stated?</br>

-Is the study design appropriate to address the stated objectives?</br>

-Is the population clearly described and appropriate for the hypothesis being tested?</br>

-Is the sample size sufficient to ensure adequate power to address the hypothesis being tested?</br>

-Were correct statistical analysis used to support conclusions?</br>

-Are there concerns about ethical or regulatory requirements being met?</br></br>

Reviewer #1: the method section is good. All the criteria were met. I just suggest to add more experiments to the paper.

Response: thanks for your comment; we have added more information on the experiments.

Reviewer #2: The objectives and study design are appropriate for the study objectives, however, more details on certain aspects of the study design are needed to assess if the experimental infections were conducted in a manner that gives accurate results. Please see the method section comments in the attached Reviewer's Comments document.

Response: thanks for your comment. Please see the revision.

Reviewer #3: The Methods are clear, I only have one question:

why were the snails fixed and not dissected alive for the trematodes? it is easier to dissect live snails

Response: Thanks for your question, we examined a total of 721 specimens of snail by molecular technique as well as dissection and it takes a lot of time to extract DNA from snails. We wanted to keep all specimens in the same condition that why we fixed all snails before dissecting them.

--------------------

<b>Results</b></br></br>

-Does the analysis presented match the analysis plan?</br>

-Are the results clearly and completely presented?</br>

-Are the figures (Tables, Images) of sufficient quality for clarity?</br></br>

Reviewer #1: The tables are fine but the figure needs better resolution

Response: Thanks, the figure has been formatted, please see the revision.

Reviewer #2: The analysis match the descriptions in the methods. The manuscript has a lot of tables and the authors may want to consider making one or two of these tables into graphs to add the reads in understanding the data.

Response: we have considered your suggestion, but we think the data are better presented in table form.

Reviewer #3: Concerning the Figure, it would be helpful to accompany it with a map of whole Vietnam and to indicate where the region under study is.

Response: Thanks for your suggestion, please see the revision.

--------------------

<b>Conclusions</b></br></br>

-Are the conclusions supported by the data presented?</br>

-Are the limitations of analysis clearly described?</br>

-Do the authors discuss how these data can be helpful to advance our understanding of the topic under study?</br>

-Is public health relevance addressed?</br></br>

Reviewer #1: Yes

Reviewer #2: Overall the discussion needs major revisions to better explain the findings and their importance as well as make the comparisons to other research more informative (instead of reading like a list of previous paper's findings). The conclusions are supported by the data, but are extended to a wider geographic area than the data support. Thus, the authors need to make revisions to the language to suggest that their finding may apply to a broader area instead of being definitive that their results are true for all locations. The discussion would be improved with a few paragraphs that explain the implications both ecologically and on human health.

Response: That is a good point and we have made text changes accordingly (see revision)

Reviewer #3: The conclusions are supported by the data, however, the authors should more emphasise the need of correct identification of parasites and hosts.

Response: thanks, please see the revision.

--------------------

<b>Editorial and Data Presentation Modifications?</b></br><br/>

Use  this section for editorial suggestions as well as relatively minor modifications of existing data that would enhance clarity. If the only modifications needed are minor and/or editorial, you may wish to recommend “Minor Revision” or “Accept”.

Reviewer #1: Please check the uploaded revised version of the manuscript.

Reviewer #2: (No Response)

Reviewer #3: The text needs a native English speaker for language checking, also, please check your text for spelling errors:

In the affiliation (line 10) please spell Vladivostok correctly

Response: Thanks for your suggestion; the language of the revised manuscript has checked by Dr. Stephen E. Greiman, Georgia Southern Univerity.

Running title – please reformulate the title, it is not understandable

Response: the running title has changed to “Identifying the first intermediate host of C. sinensis in Vietnam”

In the Introduction (line 56) for Clonorchis sinensis in the first sentence, please add the nomenclatural author

Response: the nomenclatural author is added.

line 59

please correct ‘multipleabscesses’ to ‘multiple abscesses’

Response: corrected

Table 1

it would be very helpful to provide the snail species names with nomenclatural authors and spell out in full the generic names

please correct ‘Semisulcospira libertine’ to ‘Semisulcospira libertina’

Response: thanks for your suggestion, all snail species names have added nomenclatural author. In each family, snails were arranged according to the genus. So, the first species of each genus is spelled out in full generic name, other species have the genus name abbreviated.

line 112

please correct to ‘Experimental infection of snails’

Response: corrected

line 123

please correct ‘sisnenis’ to ‘sinensis’

Response: corrected

line 129

please correct ‘tape’ to ‘tap’

Response: corrected

line 174

please correct ‘from 4 snail’ to ‘in 4 snail’

Response: I have changed this sentence “…was found from 4 snail…” to “…was released from 4 snail…”

lines 175-176

please reformulate the sentence ‘The cercariae of C. sinensis possessed only 2.85% of total

176 infected snails.‘

Response: This sentence has changed to “The cercariae of C. sinensis composed only 2.85% of cercariae from infected snails”

line 176

please correct ‘This cercariae’ to ‘These cercariae’

Response: corrected

line 193

please correct ‘infection for snails’ to ‘infection of snails’

Response: corrected

line 198

please correct ‘survive snails’ to ‘survived snails’

Response: corrected

there are several more things to be corrected in the Discussion

Response: thanks for your suggestion, please see the revison

--------------------

<b>Summary and General Comments</b></br></br>

Use  this section to provide overall comments, discuss strengths/weaknesses of the study, novelty, significance, general execution and scholarship. You may also include additional comments for the author, including concerns about dual publication, research ethics, or publication ethics. If requesting major revision, please articulate the new experiments that are needed.

Reviewer #1: PNTD-D-20-00929

Title

Are the thiarids, Melanoides tuberculata and Tarebia granifera, first intermediate hosts for Clonorchis sinensis?

This manuscript by Nguyen et al. study the potential of two thiarid snails; Melanoides tuberculata and Tarebia granifera as intermediate hosts for the Chinese liver fluke Clonorchis sinensis. They collect the snails from a parasite endemic area in Vietnam and checked it for natural infection using traditional and molecular techniques. In addition, they tried to infect the same snails from parasite free areas with C. sinensis. Based on their field data and laboratory infection experiments they concluded that these two thiarids are not potential hosts for C. sinensis and Parafossarulus manchouricus is the only molluscan intermediate host for this trematode.

General comments:

The authors did not consider that these two thiarid snails might be incompatible due to some environmental factors because they were collected from the field. Generally, it is difficult to infect field captured snails? May be they can try with their lab-bred snails.

Response: There are few publications used *natural Melanoides tuberculata* for experimental infection, e.g. Huston et al. 2018 “The life cycle of a zoonotic parasite reassessed: Experimental infection of *Melanoides tuberculata* (Mollusca: Thiaridae) with *Centrocestus formosanus* (Trematoda: Heterophyidae)”. In our experiment, we provided the same conditions for all experimental snails for comparing the infectious capacity of *C. sinensis* to each snail species. It is very difficult to lab-bred bithynids that why we decided to use natural snails which collected from parasite free area.

More experimental work can be done to detect the fate of the parasite within resistant snails such as by using histopathological sectioning of the head-foot.

Response: Absolutely, but that may be part of future studies.

A better resolution of the map is needed.

Response: done

The language of the manuscript needs some improvement.

Response: Thanks, the revision is checked carefully by Dr. Stephen E. Greiman, Georgia Southern University

Please revise the scientific names of the snails and trematode species.

Response: done

More comments and edits are included in the uploaded version of the manuscript.

Response: thank you so much for valuable comments, suggestion and corrections.

Reviewer #2: The comments in this section can also be accessed in the Reviewer's Comments document.

Dear Authors,

Overall, I found your research interesting and the multiple approaches to assess the hosts for C. sinensis quite sufficient. However, I do have a few concerns regarding the manuscript which are explained below.

Overall

•    All parts of the manuscript would be improved by changing at least half the sentences in the paragraphs to the active voice. I will provides some examples of sentences I would change to the active voice in the comments for each section.

Response: thanks for your suggestion. The revision of our manuscript is checked carefully by Dr. Stephen E. Greiman, Georgia Southern University

•    Some of the paragraphs throughout the manuscript are not indented. If this is required for the journal than nothing needs to be done, but please check to ensure that you are consistent in the use of indenting for the start of paragraphs.

Response: Please see the revision.

•    In multiple places in the manuscript, the authors assert that their findings for a single reservoir are applicable to a larger geographic area. Although their finding may suggest that the two snails are not hosts in a larger area, the wording needs to be less certain to accurately portray the research. I will provide specific comments in the section this needs to be applied, but the authors should check the entire manuscript to ensure that they are not overstating their findings.

Response: Thanks for your comments. In the introduction part, I mentioned that two thiarids have a wide distribution in the World but C. sinensis only endemic in East Asia. In general, the distribution of trematode depends on the distribution of their first intermediate hosts. In addition, I have 15 years of collecting snails and examined trematode larvae (cercariae) in Vietnam. In endemic areas of *C. sinensis*, I did not find any *M. tuberculata* and *T. granifera* specimens infected by this trematode species. From these reasons, we conducted a series experiment to verify the role of two thiarids above as the first intermediate hosts of *C. sinensis*. However, as you commented, I will add the “in Vietnam” in the title to restrict the conclusion.

Title

•    The word “Precise” in the Running Title should be replaced with a verb. For example, “Assessing” or “Evaluating” would make the Running Title easy to understand.

Response: the running title is changed to “Identifying the first intermediate host of *C. sinensis* in Vietnam”

Abstract

•    Lines 22-23:Please rewrite the second sentence to increase clarity. One possibility would be to change the sentence to read: “However, theses snails’ role as intermediate hosts has not been confirmed.” This simplifies the sentence and increases clarity.

Response: Thank you very much for improving this sentence

•    Lines 23-27: In the third sentence that begins: “Here we present” change “Here” to “Thus,” as a better transition. Also in this sentence change “firstly” to “first” and end the sentence after the word reservoir The new sentence that begins on line 27 would then read “ Next, we attempted to infect these snails with eggs of C. sinensis in laboratory experiments.”

Response: Thank you very much for your suggestion and correction, please see the revision.

•    Lines 32-34: The information on the accession numbers is out of place in the abstract. Unless required to be in the abstract by the journal, move this information to the methods and delete from the abstract.

Response: done.

•    Line 38: add “in the Thac Ba reservoir” before “in Vietnam” to make this statement more accurate as your findings only apply to this area and not the entire country.

Response: done

Authors’ Summary

•    Lines 46-47: rewrite the sentence for clarity to read “Both snail species have wide distributions in tropical and subtropical waters across the world, but C. sinensis only occurs in East Asia.”

Response: thanks for improving this sentence, please see the revision.

•    Line 48: in the second half of the sentence change “we confirmed that in Vietnam” to “confirms that in Northern Vietnam”

Response: done

•    Line 50: change “detected in Parafossarulus manchouricus by observation, molecular analyses and experimental infection”  to  “detected in a single snail species, Parafossarulus manchouricus, through observations, molecular analyses, and experimental infections.”

Response: please see the revision

Introduction

•    Line 58-60:first change “e.g.” to “including”; then remove “it from the sentence; then add “a” before “high number of human” and finally add “of infection” to the end of the sentence.

Response: done

•    Line 70: change the sentence to read “Melanoides tuberculate and T. granifera are highly invasive species and are currently present . . .”

Response: please see the revision

•    Lines 83-84: remove “e.g.” and use colon after the word “approaches” then add a comma after “experimental infection”

Response: done

Materials and Methods

•    Line 92: add an “s” to latrine

Response: done

•    Lines 93-94: place “e.g. cattle, pig, ducks, chickens, dogs, cats in parentheses

Response: done

•    Line 98: change “collecting” to “collection”

Response: corrected

•    Line 99: change “each four” to “every four”

Response: done

•    Line 112:  change “for” to “of snails”

Response: done

•    Lines 115-116: I rewrote this sentence to be in the active voice: “We collected snails from trematode-free locations and acclimatized them to laboratory conditions for seven days.” In the rest of the methods section change roughly half of the sentences to the active voice to increase readability of the manuscript.

Response: thanks for rewrote the sentence and also suggestion. Please see the revision

•    Lines 118-122: The information in this paragraph is difficult to follow. I attempted to rewrite it as follows: “For maintenance of experimental snails, we filled aquaria (plastic boxes with 2 L capacity) with dechlorinated tap water and a bottom layer of sand and small stones. To mimic the natural habitat of the snails, we add a few pieces of brushwood (Terminalia cattappa) and aquatic plants (Hygrophila difformis, Lemna minor) to each aquarium. We housed ten individuals of the same snail species in each aquarium. The number of each snail species used in this experiment varied (Table 4).

Response: thanks for improving this paragraph.

•    Line 122: The reference to Table 4 places the tables out of order. Please check the journal guidelines to determine if you need to reorder the tables to make them sequential in the manuscript.

Response: the Table 4 has rearranged.

•    Lines 123-131: The collection methods for C. sinsnesis adults and eggs need to be expanded to answer the following questions: How were cats sampled? How were eggs quantified or were large amounts just added to each aquarium? How did you ensure that eggs were present in all 5 mL allocates put into aquaria? These questions need to be addressed to better understand the experimental design and to ensure that all aquaria were actually infected with C. sinsnesis eggs.

Response: Thanks for your comments. I rewrote this paragraph, please see the revision. However, I would like to answer your questions here.

How were cats sampled? My other paper (Nguyen et al. 2018. Infections by hepatic trematodes in cats from slaughterhouses in Vietnam. Journal of Parasitology 104: 306–309) described that in cat’s slaughterhouse in Vietnam the liver and gallbladder of cats are easy collecting and we found a high prevalence and intensity infection of cats by C. sinensis.

How were eggs quantified or were large amounts just added to each aquarium*?* According to Mas-Coma & Bargues (1997) (Human liver flukes: a review. Research and Reviews in Parasitology, 57:145-218), each adult C. sinensis, which parasites in cat, produces 2400 eggs/day. We used 87 specimens of C. sinensis (amount available) for producing eggs. After this periods, we opened the uterus of each flukes to collect more eggs and then added to each aquarium. This amount of eggs ensures that the experimental snails can reach them.

How did you ensure that eggs were present in all 5 mL allocates put into aquaria? We used multichannel pipette (5 channels) with tips (1 mL) to suck the suspension. In the sucking progress, the tip point should reaches to the bottom of a glass flask which contained the suspension, then all solution in these tip were pumped into the aquarium. Of cause, amount of eggs is going down after each sucking times, however, with many eggs inside the glass flask, it will be enough for snails in the aquarium.

•    Lines 123-126: The sentences on adult flatworm collection are difficult to read – Rewrite to include answers to the previous comments’ questions.

Response: Please see the revision.

•    Lines 128-131: rewrite the sentence to “20 grams of feces from domestic cats (free from parasites) to 700 ml tap water which was shaken until the fecal matter disintegrated.”

Response: done

•    Lines 132-132: This sentence needs to be incorporated into the previous paragraph and not its own paragraph.

Response: thanks, corrected

•    Lines 151 and 156: Should these paragraphs be indented? Also, do these need to be new paragraphs?

Response: thanks for correction; I decided to divide these paragraphs because I want to separate them to three types of specimen: study specimens, positive control specimens and negative control.

Results

•    Lines 171-172: This sentence needs to be rewritten to explicitly state the C. sinensis is a pleurolophocercous cercariae. As written, it took a lot of effort including looking at the tables and search for C. sinensis on Google to determine that C. sinensis is a part of pleurolophocercous cercariae and that the authors are grouping them separately.

Response: thanks for your comments. I added two sentences “Cercariae of *C. sinensis* are belonging to pleurolophocercous group, however, these cercariae can be distinguished from others by morphological characters and theirs movement. In table 3, we arranged separately cercariae of *C. sinensis* and pleurolophocercous group” in the beginning of this paragraph

•    Lines 173-174: remove the comma after (29.21%)

Response: done

•    Lines 174-175: change “cercariae, and this type” to “which was the third most common (25.42) cercarial type.”

Response: done

•    Lines 175-176: change “infected snails. This cercariae” to “infected snails and were”

Response: done

•    Line 179: add “cercarial type” after gymnocephalous

Response: done

•    Line 185: table should be capitalized.

Response: done

•    Lines 196-197: The last two sentences in the results are difficult to read and do not make sense to me. Please rewrite these sentences for clarity.

Response: thanks for your comment, please see the revision.

Discussion

•    Overall, the discussion reads like a list of others’ findings with minimal comparisons to the finding in this manuscript. The discussion needs to be re-written to focus on the findings of this manuscript and their importance and comparison to past studies. As currently written, it is difficult to follow the reasoning or main ideas of the discussion and the conclusions are overly confident and applied to quickly to larger regions considering the research in the manuscript comes from a single reservoir. I would except more discussion on alternative explanation for why others found evidence for these two snails being the host of C. sinsnesis.

•    Given that substantial revisions of the discussion are needed, I will only point out a few other issues in this sections:

Response: thanks for your comment, please see the revision

o    Line 202: remove “the” from in front of Bithyniidae

Response: done

o    Line 205: add “distribution” after C. sinensis

Response: done

o    Line 229: change “being” to “as”

Response: done

o    Lines 231-233: this sentence is extremely difficult to understand.

Response: please see the revision

o    Line 273: C. sinensis needs to be italicized

Response: corrected

o    Lines 282-284: rewrite the sentence in the active voice: “In this study, we report the first use of 28S-rRNA gene of M. tuberculate, T. granifera, B. fuchsiana and P. machouricus form Vietnam.”

Response: done

o	Lines 285-287: This sentence is too strong of a conclusion. You did not sample locations throughout Vietnam and thus need to rewrite to be less assertive on your claims.

Response: thanks for your suggestion. I added “Northern” before “Vietnam”

Tables and Figures

•    Table 1 – In the first column, move the asterisks and ~ to after the names of the snail species to make these less distracting to the reader (unless this position in the table is required by the journal).

Response: done

•    Tables 3 and 4 --- You may want to consider making one or both of these tables into graphs to better show the data.

Response: we have considered your suggestion, but we think the data are better presented in table form.

•    Table 3 – in the footnote for this table there is a ) that seems out of place

Response: I changed the footnote, now it is similar to the footnote of Table 1.

Reviewer #3: The authors examine the ability of two snail species to host Clonorchis sinensis; it is a serious problem, as the snails are widely distributed, therefore, the paper is of importance and it is exactly within the scope of the Journal.

Most of the approach of the authors is logic, however, it is not clear already in the Abstract why they sampled also other snails and why they provide sequences of the snail hosts. This is an important addition to the topic, but it should be explained, as they did not examine only Tarebia granifera and Melanoides tuberculata.

Response: thank you very much for your comment. We were not only examining thiarid snails but also other snails in endemic area of clonorchiasis because we want to know what exactly host of *C. sinensis* in this place. We used multiple PCR approach to detect *C. sinensis* in the snails so we must provide sequences of snail hosts. However, I rewrote the abstract, please see the revision.

Also the title does not fully correspond to the contents, as it makes the reader expect that only the two snail species will be treated, however, the scope of the MS is wider.

Response: thanks for your comment. I changed the title and running title of the MS. Please see the revision

It would be wise to emphasise in the text that it is important to correctly identify the snail species and also the parasite species, as C. sinensis has pleurolophocercariae which are quite uniform in most species and they are typical in other medically important trematode families. The authors treat this in the Discussion, but it would be good to emphasise the importance of correct identification already in the Abstract, also the use of Multiplex PCR.

Response: thanks for your suggestion. Please see the revision.
