# Supplementary material for: Are Melanoides tuberculata and Tarebia granifera (Gastropoda, Thiaridae), suitable first intermediate hosts of Clonorchis sinensis in Vietnam?
Source: PLoS Negl Trop Dis. 2021 Jan 25;15(1):e0009093. doi: 10.1371/journal.pntd.0009093 (PMC7872296; doi:10.1371/journal.pntd.0009093)
Supplement: Attachment — Submitted filename: PNTD-D-20-00929.docx [file pntd.0009093.s005.docx]

PNTD-D-20-00929R1

Title

Line 2-3: for Clonorchis sinensis in Vietnam? – for instead of “of” - consider this in the entire manuscript - and in Vietnam is not italic

Abstract

Line 27-28: As well as, through experimental infections of ~~five~~ these snail species with eggs of C. sinensis. Remove five!

Line 30: Five snail species; …….. mention these species

Line 34: ~~infective~~ C. sinensis eggs…… remove infective

Line 35: infected~~, at~~ an infection rate of 7.87%. with an infection rate.

Line 38: hosts

Keywords: ~~small~~ liver fluke

Author summary

Line 45: The role that these two thiarid snail species play.

Introduction

Table 1: sometimes you put the name of the one who first described the species between brackets and sometimes not. Please unify.

Line 73: Mas-Coma & Bargues [1] mentioned that their wide distribution potentially could result in the global.

Materials and Methods
Line 113: Five snail species were chosen for experimental infection. Based on what?

Line 130: Eggs were placed in 20 g of feces.

Results

Line 165: A total of 11,985 snails, representing 10 species, were collected (Table 2). You already mentioned that in the M&M.

Line 166: snails were examined for ~~shed cercariae~~ cercarial shedding

Line 187: Table 3. Cercariae

The last column on the right No snails (%). Do you mean No of Cercariae (%)?

Line 205: Table 4. Can you make the table legend more clear? The language is not clear.

You can transfer your data to describe the snails’ survival, infection and mortality rates after 21 days of exposure to C. sinensis eggs!

Discussion

Line 227: *P. manchouricus* is a host….

Line 286: muntiplex PCR
