# Supplementary material for: Are Melanoides tuberculata and Tarebia granifera (Gastropoda, Thiaridae), suitable first intermediate hosts of Clonorchis sinensis in Vietnam?
Source: PLoS Negl Trop Dis. 2021 Jan 25;15(1):e0009093. doi: 10.1371/journal.pntd.0009093 (PMC7872296; doi:10.1371/journal.pntd.0009093)
Supplement: Attachment — Submitted filename: Response to reviewers_R2.docx [file pntd.0009093.s006.docx]

Dear Prof. Zhou and Prof. Sripa,

I and my colleagues are very happy to hear that our manuscript is likely to accept. We appreciate the reviewer insightful comments. We agree with almost all changes suggested except changing the table 3 or 4 to figure(s), and moving the first paragraph of the discussion to the introduction part. For the comments, please find our point-by-point response below clearly indicating how and where in the manuscript changes have been made. We used blue text in our response to comments.

In addition, the figure in our manuscript was uploaded to the Preflight Analysis and Conversion Engine (PACE) digital diagnostic tool, https://pacev2.apexcovantage.com. PACE and sure that this figure meet PLOS requirements.

We look forward to your decision, and hope that our manuscript is accepted to publish in PLOS NTD.

Sincerely,

On behalf of all authors,

Hung Manh Nguyen

**Responds to reviewers**:

The first reviewer

Title

Line 2-3: for Clonorchis sinensis in Vietnam? – for instead of “of” - consider this in the entire manuscript - and in Vietnam is not italic

Answer: thanks for your comment, we think “of” is more suitable than “for” in this case.

Abstract

Line 27-28: As well as, through experimental infections of ~~five~~ these snail species with eggs of C. sinensis. Remove five!

Answer: please see the revision

Line 30: Five snail species; …….. mention these species

Answer: done

Line 34: ~~infective~~ C. sinensis eggs…… remove infective

Answer: done

Line 35: infected~~, at~~ an infection rate of 7.87%. with an infection rate.

Answer: thanks, we did change this sentence.

Line 38: hosts

Answer: corrected

Keywords: ~~small~~ liver fluke

Answer: done

Author summary

Line 45: The role that these two thiarid snail species play.

Answer: the word “that” was added

Introduction

Table 1: sometimes you put the name of the one who first described the species between brackets and sometimes not. Please unify.

Answer: it is depending on each snail species which be changed to other name (synonym(s)) or not. You can check the validity of scientific name of snails in the link follow: <http://bionames.org/>

Line 73: Mas-Coma & Bargues [1] mentioned that their wide distribution potentially could result in the global.

Answer: corrected

Materials and Methods
Line 113: Five snail species were chosen for experimental infection. Based on what?

Answer: As in the introduction part, 4 of 5 snail species which using in the experimental infection were reported as the first intermediate hosts of *C. sinensis*, and all 5 species are distributed in the endemic areas of *C. sinensis* transmission.

Line 130: Eggs were placed in 20 g of feces.

Answer: Sorry for typing error, “the space” was added.

Results

Line 165: A total of 11,985 snails, representing 10 species, were collected (Table 2). You already mentioned that in the M&M.

Answer: It only mentioned in the Methodology/Principal Finding of the Abstract.

Line 166: snails were examined for ~~shed cercariae~~ cercarial shedding

Answer: corrected.

Line 187: Table 3. Cercariae

Answer: corrected

The last column on the right No snails (%). Do you mean No of Cercariae (%)?

Answer: No, it is number of snails which found to release cercariae.

Line 205: Table 4. Can you make the table legend more clear? The language is not clear.

You can transfer your data to describe the snails’ survival, infection and mortality rates after 21 days of exposure to C. sinensis eggs!

Answer: thanks for your suggestion but a lot of variables should arrange together, i.e. snail species, source of snail, source of *C. sinensis* eggs, number of snail exposed, number of snail survival, number of snail infected. That why we would like to keep this table.

Discussion

Line 227: *P. manchouricus* is a host….

Answer: corrected

Line 286: muntiplex PCR

Answer: corrected

The second reviewer

Dear Authors,

Overall, I found your manuscript much improved and easier to read, however, I still have a few concerns.

Abstract

- Lines 27-28: The sentence beginning “As well as” need to be revised to include more details. One possibility would be to rewrite the sentence as: “We also conducted experimental infection of five snail species including M. tuberculate and T. granifera with eggs of C. sinensis.”

Answer: thanks for your suggestion, the sentence was rewritten.

- Line 34: place the word “snail” between five and species to increase clarity.

Answer: done

Introduction

- Line 56: The first paragraph of the introduction needs a broader opening sentence (perhaps on the idea of trematodes as causes of human diseases or something similar)

Answer: thanks for your comment. My paper “Global status of fish-borne zoonotic trematodiasis in human. Acta Parasitologica, 2013, 58(3):231-258” and also many references mentioned very detail about trematodiasis, that why this manuscript I want to concentrate on *Clonorchis sinensis*.

- Line 60: Add a few more sentences explaining the impact these trematode infections has on humans --- this would provide the reader with a better understanding of the importance of your research.

Answer: thanks for your comment. I already coded references “Markell EK, Goldsmith R (1984) Trematode infections exclusive of Schistosomiasis. Diseases caused by liver flukes: Clonorchiasis. In: Strickland GT (ed.) Hunter’s Tropical Medicine. 6th Edition, Philadelphia, Saunders 740–755.; Rim HJ, Farag HF, Sornmani S, Cross JH (1994) Food-borne Trematodes: Ignored or Emerging? Parasitology Today 10: 207–209”, and readers will easy to find the impact of trematodes to human and animals health.

Methods

- Lines 113-117: Why were snails from Russia included in this study? You need to explain the reasoning behind adding the Russian snails since the rest of the paper focuses on Northern Vietnam.

Answer: As I mentioned in the introduction part, the range of distribution of *C. sinenis* is from Russia Far East to Northern Vietnam. In this study we used snails and *C. sinensis* samples from two border countries to find out a cross-infection among them. I add the new paragraph in the discussion part to explain about this issue, please see the revision.

- Line 123: The reference for Table 4 is out of sequence (if you refer to it here it should be Table 2 to keep the tables in sequence based on the first use in the text of the manuscript).

Answer: thanks for your comment; I deleted the word “(Table 4)”.

- Lines 124 – 129: The collection methods for *C. sinsnesis* adults and eggs need to be expanded to answer the following questions: How were cats sampled? What permits if any were used for dealing with the cats? How were eggs quantified or were large amounts just added to each aquarium? How did you ensure that eggs were present in all 5 mL allocates put into aquaria? These questions need to be addressed to better understand the experimental design and to ensure that all aquaria were actually infected with *C. sinsnesis* eggs.

Answer: thanks for a great comment, please see the revision.

- Line 130: The words Eggs and were need to be separated at the start of the sentence.

Answer: corrected

Results

- Lines 171-177: Move the sentence “A total of seven cercariae types . . .” to the beginning of the paragraph (always start with the result and then give details necessary to understand the results)

Answer: noted, thanks

- Lines 200-205: Move the section “Experimental infection of snails by C. sinensis” to line 171 and add a subheading for the molecular results (so that the subheading in the Results match and are in the same order to the subheadings used in the Methods).

Answer: thanks for your idea, however, the molecular results are belonging to two separately experiments, so we would keep the structure of our results part.

Graphs/Tables

- The captions for the tables and graphs only have single sentence titles and lack detailed explanations. These table captions and figure legend need to be expanded to be more accurate and provide more information about what is shown in the graph and tables.

Answer: According to the requirement of the PLOS NTD, the caption/legends should be concise. In addition, the important data of tables were noted in the text part.

- Table 3 or 4 could be changed to figures to make the information in these tables easier to access for the reader.

Answer: we have considered your suggestion, but we think the data are better presented in table form

Discussion

- Overall, the discussion is much improved and easier to read, but the first paragraph of the discussion would work better as the second paragraph of the introduction. By moving this paragraph to the introduction, the reader gains a better understanding of the importance of the research and why the study was conducted. You may need to rewrite a few sentences that are specific to P. manchouricus when you move this paragraph to the introduction.

Answer: thanks for your encouraged, we have considered your suggestion but we want to mention only thiarid snails in the introduction part.

- Line 246: The comma after “even though” is not necessary and needs to be deleted

Answer: done

- Line 286: The word “multiplex PCR” is misspelled

Answer: corrected

- You may want to add a sentence or two at the end of the discussion to explain the significance of the study to human health and preventing human disease due to the trematode.

Answer: we add a paragraph below before the conclusion“The results obtained can be used to clarify the list of the first intermediate hosts for *Clonorchis sinensis* throughout its range and show that in the case of studying any aspect related to trematodes, it is necessary to correctly identify not only the parasite species but also their hosts. Since wrong data incorrectly indicate the epidemiological potential of any parasite, which can lead to sudden unpredictable outbreaks of diseases in the regions. In addition, another situations possible when the spread of trematodes may be expected in the region due to erroneous identification of the parasite. In both cases, it affects the wrong investment in the development of preventive, diagnostic and therapeutic measures that contribute to the health of the population”.
