# Supplementary material for: Are Melanoides tuberculata and Tarebia granifera (Gastropoda, Thiaridae), suitable first intermediate hosts of Clonorchis sinensis in Vietnam?
Source: PLoS Negl Trop Dis. 2021 Jan 25;15(1):e0009093. doi: 10.1371/journal.pntd.0009093 (PMC7872296; doi:10.1371/journal.pntd.0009093)
Supplement: Attachment — Submitted filename: PNTD - D- 20-00929 Reviewer Comments.docx [file pntd.0009093.s002.docx]

Dear Authors,

Overall, I found your research interesting and the multiple approaches to assess the hosts for *C. sinensis* quite sufficient. However, I do have a few concerns regarding the manuscript which are explained below.

Overall

- All parts of the manuscript would be improved by changing at least half the sentences in the paragraphs to the active voice. I will provides some examples of sentences I would change to the active voice in the comments for each section.
- Some of the paragraphs throughout the manuscript are not indented. If this is required for the journal than nothing needs to be done, but please check to ensure that you are consistent in the use of indenting for the start of paragraphs.
- In multiple places in the manuscript, the authors assert that their findings for a single reservoir are applicable to a larger geographic area. Although their finding may suggest that the two snails are not hosts in a larger area, the wording needs to be less certain to accurately portray the research. I will provide specific comments in the section this needs to be applied, but the authors should check the entire manuscript to ensure that they are not overstating their findings.

Title

- The word “Precise” in the Running Title should be replaced with a verb. For example, “Assessing” or “Evaluating” would make the Running Title easy to understand.

Abstract

- Lines 22-23:Please rewrite the second sentence to increase clarity. One possibility would be to change the sentence to read: “However, theses snails’ role as intermediate hosts has not been confirmed.” This simplifies the sentence and increases clarity.
- Lines 23-27: In the third sentence that begins: “Here we present” change “Here” to “Thus,” as a better transition. Also in this sentence change “firstly” to “first” and end the sentence after the word reservoir The new sentence that begins on line 27 would then read “ Next, we attempted to infect these snails with eggs of *C. sinensis* in laboratory experiments.”
- Lines 32-34: The information on the accession numbers is out of place in the abstract. Unless required to be in the abstract by the journal, move this information to the methods and delete from the abstract.
- Line 38: add “in the Thac Ba reservoir” before “in Vietnam” to make this statement more accurate as your findings only apply to this area and not the entire country.

Authors’ Summary

- Lines 46-47: rewrite the sentence for clarity to read “Both snail species have wide distributions in tropical and subtropical waters across the world, but *C. sinensis* only occurs in East Asia.”
- Line 48: in the second half of the sentence change “we confirmed that in Vietnam” to “confirms that in Northern Vietnam”
- Line 50: change “detected in *Parafossarulus manchouricus* by observation, molecular analyses and experimental infection” to “detected in a single snail species, *Parafossarulus manchouricus*, through observations, molecular analyses, and experimental infections.”

Introduction

- Line 58-60:first change “e.g.” to “including”; then remove “it from the sentence; then add “a” before “high number of human” and finally add “of infection” to the end of the sentence.
- Line 70: change the sentence to read “*Melanoides tuberculate* and *T. granifera* are highly invasive species and are currently present . . .”
- Lines 83-84: remove “e.g.” and use colon after the word “approaches” then add a comma after “experimental infection”

Materials and Methods

- Line 92: add an “s” to latrine
- Lines 93-94: place “e.g. cattle, pig, ducks, chickens, dogs, cats in parentheses
- Line 98: change “collecting” to “collection”
- Line 99: change “each four” to “every four”
- Line 112: change “for” to “of snails”
- Lines 115-116: I rewrote this sentence to be in the active voice: “We collected snails from trematode-free locations and acclimatized them to laboratory conditions for seven days.” In the rest of the methods section change roughly half of the sentences to the active voice to increase readability of the manuscript.
- Lines 118-122: The information in this paragraph is difficult to follow. I attempted to rewrite it as follows: “For maintenance of experimental snails, we filled aquaria (plastic boxes with 2 L capacity) with dechlorinated tap water and a bottom layer of sand and small stones. To mimic the natural habitat of the snails, we add a few pieces of brushwood (*Terminalia cattappa*) and aquatic plants (*Hygrophila difformis*, *Lemna* *minor*) to each aquarium. We housed ten individuals of the same snail species in each aquarium. The number of each snail species used in this experiment varied (Table 4).
- Line 122: The reference to Table 4 places the tables out of order. Please check the journal guidelines to determine if you need to reorder the tables to make them sequential in the manuscript.
- Lines 123-131: The collection methods for *C. sinsnesis* adults and eggs need to be expanded to answer the following questions: How were cats sampled? How were eggs quantified or were large amounts just added to each aquarium? How did you ensure that eggs were present in all 5 mL allocates put into aquaria? These questions need to be addressed to better understand the experimental design and to ensure that all aquaria were actually infected with *C. sinsnesis* eggs.
- Lines 123-126: The sentences on adult flatworm collection are difficult to read – Rewrite to include answers to the previous comments’ questions.
- Lines 128-131: rewrite the sentence to “20 grams of feces from domestic cats (free from parasites) to 700 ml tap water which was shaken until the fecal matter disintegrated.”
- Lines 132-132: This sentence needs to be incorporated into the previous paragraph and not its own paragraph.
- Lines 151 and 156: Should these paragraphs be indented? Also, do these need to be new paragraphs?

Results

- Lines 171-172: This sentence needs to be rewritten to explicitly state the *C. sinensis* is a pleurolophocercous cercariae. As written, it took a lot of effort including looking at the tables and search for *C. sinensis* on Google to determine that *C. sinensis* is a part of pleurolophocercous cercariae and that the authors are grouping them separately.
- Lines 173-174: remove the comma after (29.21%)
- Lines 174-175: change “cercariae, and this type” to “which was the third most common (25.42) cercarial type.”
- Lines 175-176: change “infected snails. This cercariae” to “infected snails and were”
- Line 179: add “cercarial type” after gymnocephalous
- Line 185: table should be capitalized.
- Lines 196-197: The last two sentences in the results are difficult to read and do not make sense to me. Please rewrite these sentences for clarity.

Discussion

- Overall, the discussion reads like a list of others’ findings with minimal comparisons to the finding in this manuscript. The discussion needs to be re-written to focus on the findings of this manuscript and their importance and comparison to past studies. As currently written, it is difficult to follow the reasoning or main ideas of the discussion and the conclusions are overly confident and applied to quickly to larger regions considering the research in the manuscript comes from a single reservoir. I would except more discussion on alternative explanation for why others found evidence for these two snails being the host of *C. sinsnesis.*
- Given that substantial revisions of the discussion are needed, I will only point out a few other issues in this sections:
  - Line 202: remove “the” from in front of Bithyniidae
  - Line 205: add “distribution” after *C. sinensis*
  - Line 229: change “being” to “as”
  - Lines 231-233: this sentence is extremely difficult to understand.
  - Line 273: C. sinensis needs to be italicized
  - Lines 282-284: rewrite the sentence in the active voice: “In this study, we report the first use of 28S-rRNA gene of *M. tuberculate, T. granifera, B. fuchsiana* and *P. machouricus* form Vietnam.”
  - Lines 285-287: This sentence is too strong of a conclusion. You did not sample locations throughout Vietnam and thus need to rewrite to be less assertive on your claims.

Tables and Figures

- Table 1 – In the first column, move the asterisks and ~ to after the names of the snail species to make these less distracting to the reader (unless this position in the table is required by the journal).
- Tables 3 and 4 --- You may want to consider making one or both of these tables into graphs to better show the data.
- Table 3 – in the footnote for this table there is a ) that seems out of place
