# Supplementary material for: Are Melanoides tuberculata and Tarebia granifera (Gastropoda, Thiaridae), suitable first intermediate hosts of Clonorchis sinensis in Vietnam?
Source: PLoS Negl Trop Dis. 2021 Jan 25;15(1):e0009093. doi: 10.1371/journal.pntd.0009093 (PMC7872296; doi:10.1371/journal.pntd.0009093)
Supplement: Attachment — Submitted filename: PNTD - D- 20-00929_R1 Reviewer Comments.docx [file pntd.0009093.s004.docx]

Dear Authors,

Overall, I found your manuscript much improved and easier to read, however, I still have a few concerns.

Abstract

- Lines 27-28: The sentence beginning “As well as” need to be revised to include more details. One possibility would be to rewrite the sentence as: “We also conducted experimental infection of five snail species including M. tuberculate and T. granifera with eggs of C. sinensis.”
- Line 34: place the word “snail” between five and species to increase clarity.

Introduction

- Line 56: The first paragraph of the introduction needs a broader opening sentence (perhaps on the idea of trematodes as causes of human diseases or something similar)
- Line 60: Add a few more sentences explaining the impact these trematode infections has on humans --- this would provide the reader with a better understanding of the importance of your research.

Methods

- Lines 113-117: Why were snails from Russia included in this study? You need to explain the reasoning behind adding the Russian snails since the rest of the paper focuses on Northern Vietnam.
- Line 123: The reference for Table 4 is out of sequence (if you refer to it here it should be Table 2 to keep the tables in sequence based on the first use in the text of the manuscript).
- Lines 124 – 129: The collection methods for *C. sinsnesis* adults and eggs need to be expanded to answer the following questions: How were cats sampled? What permits if any were used for dealing with the cats? How were eggs quantified or were large amounts just added to each aquarium? How did you ensure that eggs were present in all 5 mL allocates put into aquaria? These questions need to be addressed to better understand the experimental design and to ensure that all aquaria were actually infected with *C. sinsnesis* eggs.
- Line 130: The words Eggs and were need to be separated at the start of the sentence.

Results

- Lines 171-177: Move the sentence “A total of seven cercariae types . . .” to the beginning of the paragraph (always start with the result and then give details necessary to understand the results)
- Lines 200-205: Move the section “Experimental infection of snails by C. sinensis” to line 171 and add a subheading for the molecular results (so that the subheading in the Results match and are in the same order to the subheadings used in the Methods).

Graphs/Tables

- The captions for the tables and graphs only have single sentence titles and lack detailed explanations. These table captions and figure legend need to be expanded to be more accurate and provide more information about what is shown in the graph and tables.
- Table 3 or 4 could be changed to figures to make the information in these tables easier to access for the reader.

Discussion

- Overall, the discussion is much improved and easier to read, but the first paragraph of the discussion would work better as the second paragraph of the introduction. By moving this paragraph to the introduction, the reader gains a better understanding of the importance of the research and why the study was conducted. You may need to rewrite a few sentences that are specific to P. manchouricus when you move this paragraph to the introduction.
- Line 246: The comma after “even though” is not necessary and needs to be deleted
- Line 286: The word “multiplex PCR” is misspelled
- You may want to add a sentence or two at the end of the discussion to explain the significance of the study to human health and preventing human disease due to the trematode.
